# Patient Perceptions of Blockchain-Based Health Information Exchange: User-Centered Design Study

**DOI:** 10.2196/78849

**Published:** 2026-03-11

**Authors:** Richard Guse, Shanshan Hu, Scott Thiebes, Christina Erler, Catherine Caridia, Wilhelm Stork, Martin Gersch, Ali Sunyaev

**Affiliations:** 1 Department of Economics and Management, Karlsruhe Institute of Technology, Kaiserstraße 89, Karlsruhe, 76133, Germany, 49 721 608-43679; 2 School of Computation, Information and Technology (CIT), Technical University of Munich, München, Germany; 3 Advanced Institute of Business, School of Economics and Management, Tongji University, Shanghai, China; 4 FZI Research Center for Information Technology, Karlsruhe, Germany; 5 Charité Comprehensive Cancer Center, Charité - Universitätsmedizin Berlin, Berlin, Germany; 6 Department of Information Systems, School of Business and Economics, Freie Universität Berlin, Berlin, Germany

**Keywords:** electronic health records, blockchain, patients, user-centered design, acceptance, usability, technology acceptance model, System Usability Scale, surveys and questionnaires

## Abstract

**Background:**

Blockchain-based health information exchange (HIE) has received increased attention in health care research and practice over the last years. It enables the sharing of patient information across healthcare organizations, provides higher levels of data confidentiality and security, and reduces time and costs in collaborative medical decision-making. To make informed decisions on the implementation of blockchain-based HIE in practice and to fully understand the implications of its use for patient care, it is important to gain insights into patient perceptions of and interactions with blockchain-based HIE.

**Objective:**

This study aimed to assess patient perceptions of a blockchain-based HIE mobile app to inform its iterative development.

**Methods:**

We used a mixed methods user-centered design in 3 phases to iteratively assess patient perceptions of blockchain-based HIE: (1) structured questionnaires collecting patient requirements for blockchain-based HIE, (2) semistructured interviews evaluating mobile app mock-ups, and (3) a survey with blockchain-based HIE scenarios related to their patient care using the technology acceptance model, System Usability Scale, and open feedback. Both the semistructured interviews and the survey were conducted in a clinical setting with patients with cancer undergoing treatment at a major university hospital in Germany. As an exemplary case, we deem patients with cancer as well-positioned to evaluate a blockchain-based HIE mobile app since their treatment requires extensive coordination and data sharing across health care providers.

**Results:**

Our findings support that patients have a high intention to use the blockchain-based functions that enable them to define, track, and revoke access to their health data per health care facility and service provider. Patients rated the 4 key functionalities (connection with providers, document sharing, a health diary, and a health care service provider search) as both useful and easy to use. The overall System Usability Scale of the blockchain-based HIE mobile app improved over the 3 phases up to 77.34, showing a good overall usability. The open feedback showed that patients’ perceived usefulness of a blockchain-based HIE mobile app is especially influenced by 3 factors: the acceleration of the process of data sharing, patient-centered access control, and alignment with the respective health care settings. Moreover, patients’ perceived ease of use of a blockchain-based HIE mobile app is impacted by 3 additional factors: the intuitiveness of the interaction, an aesthetic and functional design, and individual differences such as age or literacy with document management systems.

**Conclusions:**

The evaluation demonstrates that patients are inclined to use blockchain-based HIE to manage their health data, as it empowers them to control which health care providers or individuals can access their information. To foster the use of a blockchain-based HIE mobile app, the app should allow patients to effortlessly establish connections with health care providers, offer an overview of all patient data, and enable patients to share medical documents individually via the app.

## Introduction

### Background

Patients often receive care from multiple health care providers throughout their lifetime, leading to health data being fragmented across institutions [1,2]. Multiple mechanisms have been introduced to address such fragmentation, including manual data transfers (eg, on paper or digital storage media) [3], electronic transfers via health information exchanges (HIEs) [4,5], and efforts aimed at centralizing patient information through general practitioners (eg, in the United Kingdom [6] or Germany [7]) or in regional or national databases (eg, in the United Kingdom [8] or Denmark [9]). Yet, patient data fragmentation persists [5,10], especially due to challenges such as incomplete records [6], data confidentiality concerns [11], and security vulnerabilities [12,13]. As a result, clinicians frequently lack a complete view of patients’ medical histories, contributing to redundant tests, delayed diagnoses, and higher health care costs [1,14]. Developing a fully integrated, real-time patient record, therefore, remains a major challenge.

Blockchain technology has recently emerged as a promising solution for addressing data fragmentation issues and building interoperable, patient-centered HIEs [15,16]. By providing a decentralized, tamper-resistant ledger for managing consent and data access, blockchain can enhance data security and privacy [3,17] and give patients greater control over their information [17]. Especially, it enables patients to have an up-to-date overview of their health data (eg, which data are stored by which health care providers) and to decide independently who (eg, health care providers or service providers) can access their data and for what purposes [16]. These properties have positioned blockchain-based HIE as one of the most frequently discussed use cases of blockchain in health care among researchers and practitioners [15-20]. However, broad adoption of blockchain-based HIE still remains an issue. Reasons for this issue include a lack of organizational commitment, the absence of real-world testing of blockchain benefits in health care, and the functions of BC-based HIE being intangible for patients [19].

Prior research on blockchain-based health data management has examined diverse aspects, including challenges and benefits of its application [16,21-23], technical specifications [20,24,25], and users’ attitudes toward different blockchain-based HIE functionalities [3,15,26,27]. These studies generally indicate that both health care professionals and patients have a positive attitude toward blockchain-based HIE and recognize its potential for improving transparency and data control. However, most of these investigations have focused on measuring general attitudes or perceived benefits through hypothetical scenarios [3,27,28] or interview-based discussions [15,29]. Thus, they mainly capture anticipated advantages rather than experienced ones. Only a few studies have involved prototype evaluations. Those studies typically focus on isolated functions, such as data sharing with research organizations [28], transaction tracking [30], or consent management [31], without allowing patients to actively manage access rights and view provider-specific medical records within an integrated system environment. Moreover, many blockchain-based HIE apps are still in a conceptualization stage, with very few having progressed to prototype development, let alone to actual implementation stages [16]. Consequently, a notable research gap remains regarding empirical evidence on how patients actually experience and interpret blockchain-based HIE in practice.

### Objectives of the Study

Building on the identified research gap regarding the limited empirical evidence on how patients experience and interpret blockchain-based HIE systems in practice, this study aims to advance the understanding of patient engagement with such technologies beyond hypothetical attitudes. To this end, we conducted a 3-phase, user-centered design and evaluation of a functional blockchain-based HIE prototype, where patients recruited from a major university hospital interact with realistic usage scenarios (ie, patient-facing, practice-oriented interactions). Specifically, the study pursues three objectives: (1) to report on the user-centered design of a blockchain-based HIE mobile app prototype that enables patient-controlled management and sharing of health data; (2) to assess patients’ experiences of blockchain-based HIE related to their current treatment in a clinical context and to evaluate their perceptions of the prototype’s usability, usefulness, and overall user acceptance across four realistic HIE scenarios; and (3) to derive implications for the user-centered design and adoption of blockchain-based HIE solutions by identifying key functionalities and design features that influence user acceptance.

By examining patients’ lived interactions with a functional blockchain-based HIE mobile app, this study provides empirical insights into how blockchain functionalities shape user experiences and adoption intentions in health care contexts. With our focus on evaluating a functional blockchain-based HIE mobile app with real patients, we explicitly go beyond the analysis of hypothetical attitudes as reported in prior research [3,27,28]. In addition, by examining patients’ perceptions related to usability, usefulness, and acceptance across different functions of a blockchain-based HIE app, our study identifies which functions are crucial for blockchain-based HIE applications and where design improvements are needed. This is especially relevant given that blockchain-based applications are often characterized by low usability, as users have to invest a considerable effort to learn how to use these applications (eg, by installing and managing wallets for private keys) [32,33]. These insights can help researchers and practitioners identify barriers to the adoption of blockchain-based HIE, predict its actual use in the future, and make informed user-centered decisions about its implementation [34,35].

## Methods

### Overview

The development of the blockchain-based HIE app was carried out in collaboration with public health care providers and service providers (eg, a health cloud provider) in the context of a collaborative research project, BloG3. Our aim was to develop and test a blockchain-based HIE mobile app in the hospital context that would support patients in retrieving and sharing documents from service providers and health care providers. For the development of the blockchain-based HIE mobile app, we used user-centered design involving patients as primary users to cater the mobile app to patients’ needs [36,37]. User-centered design provides guiding principles and suggests techniques at various stages of the design process [36]. It ensures that an app is easy to learn, offers intuitive interactions, and is tied to user requirements. As our main study site, we chose the oncology department of a major university hospital in Germany, from where we recruited patients with cancer to evaluate the mobile app. We chose patients with cancer as the primary user group since their complex treatment pathways demand extensive coordination and data sharing across health care providers. In addition, their physical and emotional challenges further make usability especially critical for an HIE application. These characteristics make patients with cancer a crucial test case for evaluating blockchain-based HIE, which aims to support secure, patient-centered data sharing in contexts that require high coordination.

**Figure 1.**
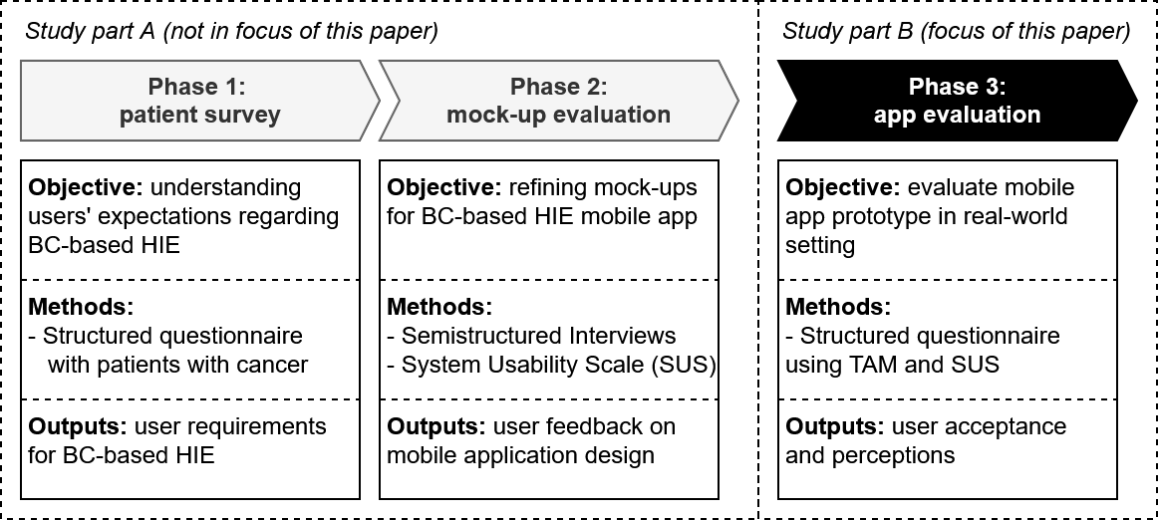
Phases of the development and evaluation process for the blockchain (BC)-based health information exchange (HIE) mobile app. TAM: technology acceptance model

The research project was divided into 3 phases in which we iteratively developed and evaluated the functionalities of the mobile app (refer to Figure 1). The 3 phases enabled us to incorporate patient needs from an early design stage to the full implementation of the mobile app. After identifying user requirements (phase 1), we developed a first design of the mobile app (mock-ups) based on the identified user requirements. Subsequently, we evaluated the mock-ups with patients to receive further user feedback on the design (phase 2). Finally, we implemented a blockchain-based HIE mobile app and collected user perceptions of the functionalities of the mobile app in a clinical context, with patients undergoing medical treatment in a hospital, realistic usage scenarios, and realistic sample documents related to their care (phase 3). While phases 1 and 2 yielded important information for the development of the mobile app, phase 3 served to provide us with comprehensive feedback on the usability, usefulness, and acceptance of the implemented mobile app. This feedback is especially helpful to determine barriers to the adoption of blockchain-based HIE (eg, low usability) and predict its actual use in the future. Hence, while we also report on the results of phases 1 and 2, we put a higher focus on reporting the results of phase 3 in this study.

### Phase 1: User Requirements Elicitation

In the first phase, we identified user requirements (eg, preferred app features). The aim of this phase was to get an overview of important features and health data that should be included in the blockchain-based HIE mobile app. To identify user requirements, we devised an online survey with questions about patients’ current overview of their health data, whether they want to manage access rights per facility and doctor, whether they would like to have an overview of who accessed their health data, which documents they deem relevant to share, and which device they would like to use to manage their health data. Questions were iteratively developed by the author team based on prior literature on blockchain-based HIE [4,5,5-16], in a structured format, and could be answered on a 6-point Likert scale ranging from “1 (strongly disagree)” to “6 (strongly agree).” For pretesting the understandability of the questionnaire, we recruited a convenience sample of 18 participants who provided us with feedback, which we used to clarify unclear descriptions and specify ambiguous wording. An overview of the questionnaire and the survey results is available in Multimedia Appendix 1. We recruited participants via oncology patient communities nationwide by mail, inviting them to participate in the online survey. Patients with cancer in treatment or aftercare, next of kin, and other noncancer patients (eg, significant others) were able to participate to collect a broad overview of groups involved in the management of patient documents throughout the care process.

### Phase 2: Mock-Up Evaluation

**Figure 2.**
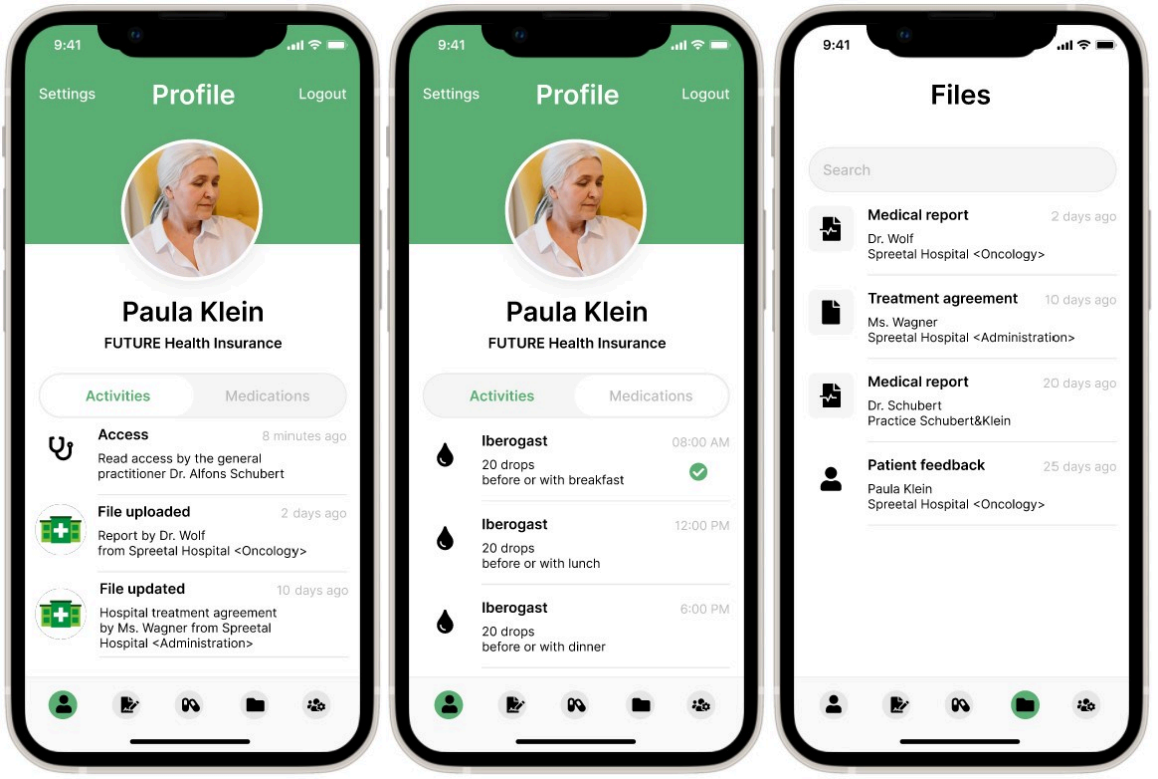
Mock-up screens of the blockchain (BC)-based health information exchange (HIE), showing a general overview of recent patient document interactions, medications, and available patient documents.

In the second phase, the identified user requirements were used as a foundation to develop mock-ups for a blockchain-based HIE mobile app consisting of a total of 36 clickable frames on a handheld computer. We developed the mock-ups iteratively within the author team with external partners experienced in mobile app development for health care use cases. We evaluated the mock-ups with semistructured interviews during which we collected patient impressions of their interaction with the clickable mock-ups. Patients were asked to access different functionalities in the mock-ups, which were chosen based on user feedback from phase 1 and to answer questions related to the mock-ups. The functionalities included an overview of patient documents and medications, a diary for patient mood and health condition, and an overview of documents shared with exemplary health care facilities. An overview of the mock-up screens is shown in Figure 2. Further screens are available in Multimedia Appendix 2. The questions were developed based on the questionnaire from phase 1 and refined by the author team. These included questions related to the overall impression of the blockchain-based HIE mobile app, whether they would use it, and which medical documents they attach the greatest importance to.

To measure usability, we collected patient perceptions of the app using the System Usability Scale (SUS) using an established German translation [38]. We chose SUS as it is established for measuring the user experience of a given software application [39]. We classify the SUS with an established grading scale ranging from a worst grade of “F” SUS range of 0.0‐51.6 to a best grade of A+ range of 96.0‐100 to assess the usability with a measure that shows what an acceptable SUS score is [39]. The interviews took place on-site in the oncological outpatient department of our main study site. We recruited adult patients at random, by willingness to participate, through on-site research staff. We applied quota sampling by time to randomize participants; that is, we recruited patients on several days at different times. Due to ethical concerns, we explicitly excluded patients younger than 18 years. We emphasized to patients that refusing to participate would not affect their treatment in any way. If patients refused to participate, we did not contact them again and excluded them from data collection. A researcher of the author team introduced patients to the idea of the blockchain-based HIE mobile app, observed patients while they clicked through the mock-ups, and gave instructions if needed. After patients finished accessing all functionalities, they filled out the SUS on a paper sheet. We aimed to provide only minimal guidance for clicking through the mock-ups. We encouraged participants to explore the functionality of the app themselves on a provided mobile device. If a participant could not access certain functionality, the researcher showed them how the functionality could be accessed by clicking through the mock-ups. While the researcher focused on highlighting the functionality of the mock-ups, BC-based HIE was introduced to patients as the mechanism that enabled sharing and revoking access to patient documents.

### Phase 3: Mobile App Evaluation

In the final phase, we developed the blockchain-based HIE mobile app and a blockchain-based backend infrastructure, which enabled patients to share their medical documents. While our system was based on a system design recently proposed in the literature [40], we based the mobile app on the clickable frames from phase 2. To evaluate the blockchain-based mobile app, we conducted interviews with patients in the oncological outpatient department of our main study site using the mobile app in practice-oriented scenarios that users face during their treatment.

### Description of the Developed Blockchain-Based HIE System

**Figure 3.**
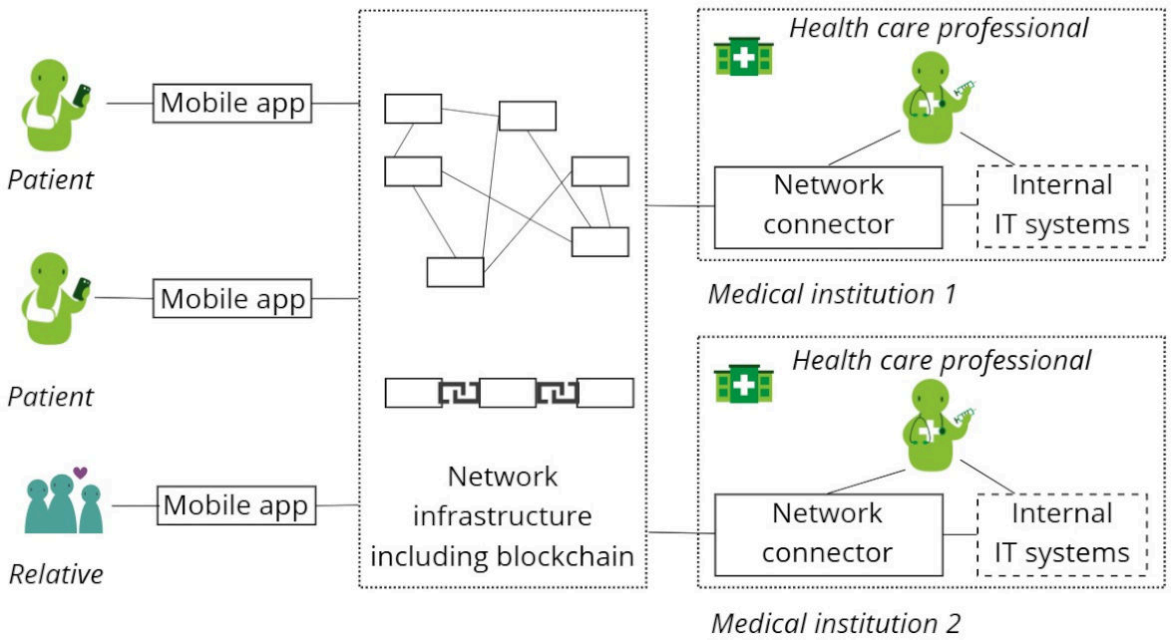
Blockchain (BC)-based health information exchange (HIE) system overview. IT: Information Technology

For our developed blockchain-based HIE system, we adopted a system design recently proposed in the literature [40]. During system design, we incorporated threat modeling using the STRIDE (Spoofing, Tampering, Repudiation, Information disclosure, Denial of service, Elevation of privilege) method to systematically identify potential security risks, inform the architecture, and ensure secure interorganizational health data exchange [41]. An overview of the system is shown in Figure 3. The system consisted of the following four components: (1) a mobile app, (2) network infrastructure with a blockchain, (3) a connector to the IT systems of health care facilities and service providers, and (4) the existing internal IT systems at the health care facilities and service providers.

The network infrastructure is a decentralized platform secured by blockchain technology. It enables secure, asynchronous, encrypted peer-to-peer communication and data exchange between patients’ mobile apps and connectors of health care facilities and service providers. The blockchain publishes a secure and tamper-proof registry for digital signatures with which patients, health care facilities, and service providers verify their identities. This enables patients to grant access rights to their health data to trusted health care facilities, service providers, and individuals based on their verified identity. Health data are stored off-chain within the existing infrastructure of the health care facility and service providers, ensuring that the data remain securely within the provider’s systems, thereby eliminating the need to rely on external systems for trust. Additionally, there are no issues with deleting health data from the blockchain, which is a common challenge for blockchain use in health care. The connector is integrated into the internal IT systems of health care facilities and service providers and acts as an interface within the network infrastructure. This interface facilitates seamless access to data stored within health care facilities and service providers while ensuring interoperability and compliance with privacy and security standards. The system is based on the open-source implementations of Hyperledger Aries [42] and Indy [43] by the LF Decentralized Trust as part of the Linux Foundation, with connectors implemented using Aries and the public blockchain using Indy. Hyperledger Indy serves as the blockchain-based registry for identity management and credential verification. For this, Indy uses Plenum, an implementation of the Redundant Byzantine Fault Tolerance consensus mechanism. It should be noted that during the evaluation, no connection to live EHR systems or national health infrastructures was implemented. However, we set up connectors at 2 different health care facilities, including our main study site, to provide synthetic patient data during the evaluation. Overall, the system architecture was designed to be modular and extensible to allow future adaptations.

### Description of the Mobile App for Patients

**Figure 4.**
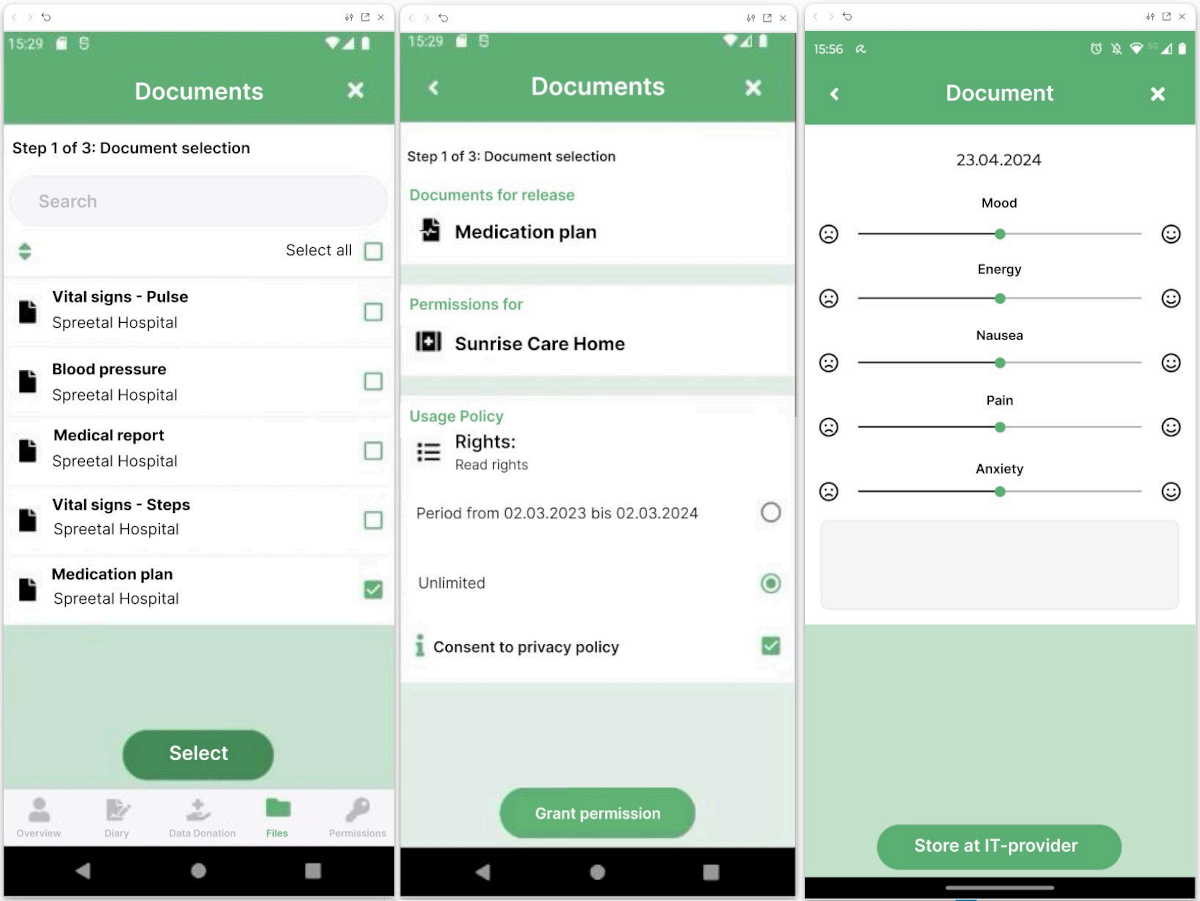
Blockchain-based health information exchange (HIE) app screens for sharing specific documents with health care and service providers and entering data into a patient diary.

The mobile app is the user interface of the developed blockchain-based HIE system for patients and was developed based on the patient requirements from phase 1 and the feedback to the mock-ups from phase 2. It serves as a central access point and hub for the management of patients’ health records stored at different health care facilities and service providers. By actively involving patients, the mobile app ensures privacy and control over their health data. Moreover, the mobile app was developed using the Xamarin platform to enable cross-platform deployment [44]. The 4 key functionalities of the mobile app include establishing a connection with a health care or service provider by scanning a QR code, selecting and sharing specific documents through the system with other health care and service providers involved in patient care (Figure 4), creating health diary entries, and searching for nursing homes within a given area. An overview of the blockchain-based app screens is included in Multimedia Appendix 3.

For patients to access their health data, a connection to the health care facility or service provider is required. Establishing a connection begins with the patient scanning a QR code provided by the health care or service provider using their mobile app. This action initiates the registration of the patient’s mobile app with the service provider’s connector. Subsequently, the service provider is listed as a registered contact in the patient’s mobile app. When new health data becomes available, the patient receives an automatic notification via their mobile app. The patient can directly access and filter the health data from the service provider using the mobile app. Furthermore, they can grant other registered organizations access to this data. Patients can share their data from the primary health market, like health care providers (eg, findings and medical reports), data from the secondary health care market, like service providers (eg, recordings of fitness watches), as well as self-generated patient-reported outcome data (eg, symptoms in a patient diary). The patient-reported outcome data can be documented multiple times a day using a diary function with 5 entry types that can be recorded using sliders: Mood, Energy, Nausea, Pain, and Anxiety. A free-text field was included for individual comments. Based on patient feedback during interactions with the mock-ups, an additional feature was implemented, enabling patients to search for nursing homes within a specified area. This function was added since patients wanted to search and connect to further relevant providers directly via the mobile app without the need for scanning another QR code. Overall, to further improve the usability of the app, we added more information to symbols and buttons since patients indicated that the symbols used were not intuitive.

### Study Description for Evaluating the Mobile App

For the evaluation of the blockchain-based HIE mobile app, we recruited patients from the oncological outpatient department of our main study site. A researcher from the author team recruited adult patients based on their willingness to participate. We randomized participants by applying quota sampling by time. Accordingly, we recruited patients on several days at different, varying time slots, thereby reducing time-based recruitment bias. Systematic randomization was not applicable since the time for data collection was limited. We explicitly excluded patients younger than 18 years due to ethical concerns. We informed patients that their participation in the study was voluntary and would not affect their care in any way. When patients refused to participate, we did not contact them any further and excluded them from further data collection. For the systematic evaluation of the blockchain-based HIE mobile app, we devised a questionnaire that introduced the participants to blockchain-based HIE, asked for demographic information, and asked the participants to complete 4 tasks with the mobile app. The questionnaire was developed and assessed in several iterations within the author team to improve its understandability. To ensure its suitability for measuring patient perceptions, we revised the questionnaire in terms of understandability and clarity based on the feedback from 2 IT professionals of the collaborative research project BloG3 in which the studies were situated and 2 physicians. The questionnaire is available in Multimedia Appendix 4, and an overview of the survey results is available in Multimedia Appendix 5.

While the researcher on-site presented the questionnaire to patients on paper, the blockchain-based HIE mobile app was handed out on a provided mobile device to patients. Similar to phase 2, the researcher offered only limited guidance for clicking through the mobile app. The researcher waited at least 30 seconds until a participant received help, encouraging participants to complete the questionnaire by exploring the functionality of the mobile app themselves. If participants indicated that they did not wish to proceed with a task, the researcher explained to the participant where to click.

The first part of the questionnaire contained a brief introduction to the concept of blockchain-based HIE. The key functionalities of the blockchain-based HIE were highlighted as the scanning of a unique decentralized identifier (DID) via a QR code and the function of sharing and revoking access to patient documents. Patients could only perceive the functionality that is enabled and to some degree necessitated by using a blockchain (eg, when establishing a connection with a health care facility or service provider). Next, we collected demographic information on gender, age range, number of document management systems used (eg, cloud storage providers), and frequency of their use.

The main part of the questionnaire set 4 tasks that patients had to complete with the mobile app. Each task relates to 1 of the 4 key functionalities of the mobile app. For each task, we collected 2 outcome measures by noting whether a patient was able to complete a task without assistance and asking patients to confirm the result of a task (eg, being connected to a specific health care facility). If a patient indicated having difficulties completing a task, the researcher assisted the patient in completing the task on the mobile device by providing information on what to look for. After completing a task, patients were asked to assess the functionality with the 10-item questionnaire of the technology acceptance model (TAM) [45]. The items measured the 3 main TAM constructs, perceived usefulness (PU), perceived ease of use (PEOU), and behavioral intention (BI) to use, on a 7-point Likert scale ranging from “1 (strongly disagree)” to “7 (strongly agree).” The TAM constructs enable the measurement of user perceptions of how useful an application is for a given task (PU), how effort-free the interaction is (PEOU), and how likely they will adopt an application [45]. We chose TAM as it is an established measurement tool to evaluate user perceptions of software applications that allow us to predict whether users are likely to use an app before its rollout [46]. For our study, we based the items on a validated German translation [47]. In task 1, patients were asked to establish a connection with a health care facility by scanning a QR code. Task 2 requested patients to share preset medical documents and health data (ie, a medication plan, blood pressure values, heart rate, and steps) in their mobile app with a health care facility and a service provider. For one, they shared the medical document for a limited timeframe. For the other, they were required to share and revoke access to the medical document. In task 3, patients were asked to enter a random mood and health condition, share it with one of the health care facilities or service providers, and delete it. In task 4, patients searched for a nursing home to which their health data could be shared via the mobile app to support the discharge process. After completing all tasks, patients were asked to evaluate the overall usability of the blockchain-based HIE mobile app with the SUS [38]. Similar to phase 2, we classify the SUS with an established grading scale [39]. Finally, we collected free-text responses to get additional qualitative feedback on perceived advantages, challenges of the app, and suggestions for improvement.

### Data Analysis

For every task, we calculated the mean scores and SDs for the used constructs (PEOU, PU, and BI). In addition, we checked the scale reliability of the used constructs, since we reworded the items to include the name of the tested blockchain-based HIE mobile app instead of a general “system.” We thereby ensure that the items work cohesively for our study setting. For testing the scale reliability, we checked the internal consistency of the used, reworded items for PEOU and PU with Cronbach α [48]. For measuring the internal consistency of the items for the construct BI, we used the Spearman-Brown coefficient, since BI is only a 2-item scale for which Cronbach α cannot adequately determine the reliability [49].

**Figure 5.**
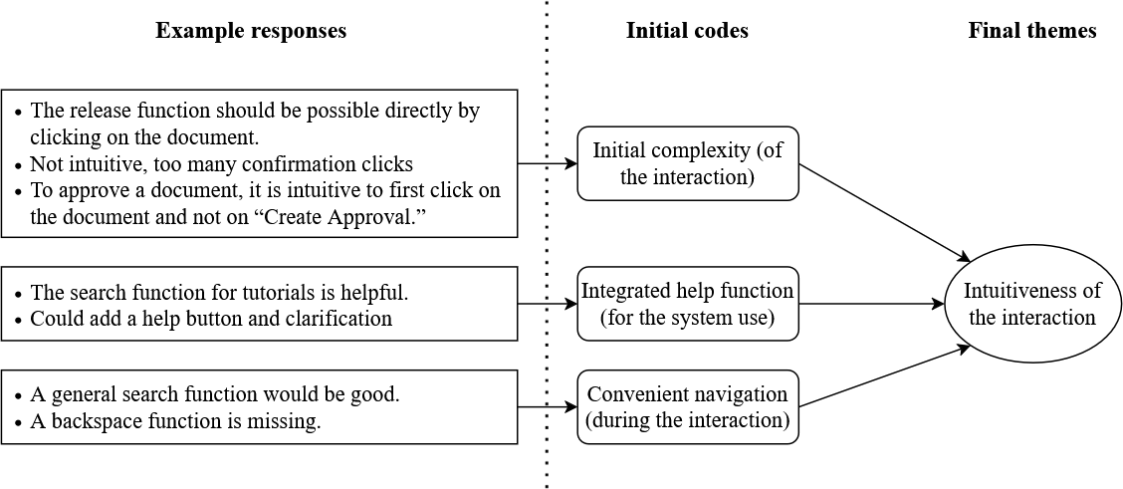
Excerpt from the qualitative coding process.

To better understand the rationale behind participants’ perceptions, we applied thematic analysis to patient responses to the 3 open questions. For the analysis, we followed the 6 phases outlined in [50]. In the first phase (familiarizing with the data), 2 authors independently reviewed the responses and mapped each to 2 of the 3 TAM constructs that influence the intention to use (ie, PU and PEOU). During the mapping process, they extracted key statements describing factors influencing these 2 constructs and performed an initial round of coding to generate first-order codes (second phase: generating initial codes). The 2 authors synthesized similarities and discussed differences among these initial codes with 1 additional author. In the third phase (searching for themes), 2 authors iteratively identified relationships between initial codes and clustered them into broader, conceptually connected themes. In the subsequent phase (reviewing themes), the team evaluated the internal consistency and distinctiveness of these candidate themes, ensuring that each accurately reflected the coded data and the dataset as a whole. This stage involved integrating closely related themes, subdividing those that were too encompassing, and reallocating codes that did not clearly belong to their original theme. This iterative process led to the identification of 6 themes. In the fifth phase (defining and naming themes), we defined the themes by identifying the “essence” of each theme and clarifying its conceptual boundaries (ie, what aspect of participants’ responses it represented and what it excluded). Based on their definitions, we named the refined themes and categorized them based on their contextual dependencies. The final outcomes of this analysis are detailed in the “Results” section (phase 6: producing the report). An illustrative example of this process is provided in Figure 5.

### Ethical Considerations

All study parts were approved by the local ethics committee (Ethikkommission der Charité, no EA4/241/21). In the online survey in phase 1, a privacy statement informed participants about how their data were used and processed for the study. In phases 2 and 3, patients provided informed consent for the interviews. In all study parts, participation was voluntary and anonymous.

## Results

### Phase 1: Collection of User Requirements

In phase 1, we collected 108 responses from potential users, of which 60 completed the whole questionnaire (31 patients with cancer and 29 significant others involved in their care). From these, we could derive 4 requirements for a blockchain-based HIE application. An overview of participants’ gender and age distribution is shown in Table 1. The responses showed that participants rather agreed with having a complete overview of their current health documents, although they indicated that neither their primary care physicians nor their relatives have a complete overview. More importantly, half of the participants (n=30) stated that they forgot or lost documents themselves, their health care staff lost documents, or documents were provided too late. Hence, we defined as the first requirement that the application should contain an overview of patients’ health documents (Req 1). Regarding the platform for managing health data, participants stated that both a computer and a smartphone would be their preferred way for managing their health data. To allow for better portability and easier access everywhere, we decided to focus on a mobile app and defined the second requirement: the app should be accessible via smartphone (Req 2). Of all possible medical documents and health data, participants rated diagnosis reports, laboratory results, imaging results, and their medication plan as most important for sharing them with their health care providers. Thus, the app should at a minimum support the sharing of diagnosis reports, laboratory results, imaging results, and their medication plan (Req 3). Participants displayed high agreement with whether they would like to define who can access, edit, and delete their data per health care facility and physician. Participants preferred sharing specific documents instead of all data at once. In addition, they gave high priority to being able to track and revoke who accesses their data. Hence, the application should enable users to share, edit, track, and delete access rights to their medical documents per document per healthcare facility or service provider (Req 4). Overall, participants supported the idea of a blockchain-based HIE mobile app to manage access to their health data.

**Table 1.** Demographic data of participants for phase 1 (N=60).

Participant demographic	Values (N=60), n (%)
Sex	
Diverse	1 (1.5)
Female	28 (41.8)
Male	31 (56.7)
Age (years)	
18‐29	9 (15)
30‐39	13 (21.7)
40‐49	7 (11.7)
50‐59	13 (21.7)
60‐69	13 (21.7)
70‐79	4 (6.7)
80‐89	1 (1.7)

### Phase 2: Mock-Up Evaluation

In phase 2, we recruited 22 patients with cancer to evaluate the mock-ups of the blockchain-based HIE mobile app. Seventeen patients declined to participate. An overview of participants’ gender and age distribution is shown in Table 2. The semistructured interviews provided insights into patient perceptions of the mobile app. Overall, the expectations of interviewed patients were positive. Patients stated that they would be very likely to use the mobile app once it is fully implemented. Patients had high anticipation of more efficient and transparent management of medical health care documents as well as faster communication between all stakeholders involved in the treatment process. The responses indicated that patients attach great importance to being able to manage the following health data in a blockchain-based HIE mobile app: medical findings, diagnoses, operating reports, laboratory results, and the medication plan.

**Table 2.** Demographic data of participants for phase 2 (n=22).

Participant demographic	Value, n (%)
Sex	
Female	14 (63.6)
Male	8 (36.4)
Age (years)	
18‐29	0 (0)
30‐39	3 (13.6)
40‐49	4 (18.2)
50‐59	8 (36.4)
60‐69	6 (27.3)
70‐79	1 (4.5)

For the clickable mock-ups, patients rated the usability with the SUS on average as 66.50, which hinted that the usability is satisfactory and can be graded as a “C” [39,51]. Still, several participants hinted that there may be challenges for older people to use the mobile app since several clicks were necessary to access certain functionalities. This challenge was noted and accounted for in the next phase of development to further enhance the usability of the mobile app.

### Phase 3: Mobile App Evaluation

#### User Statistics

In the last phase, 32 participants were recruited, consisting of 13 males and 19 females. Nineteen patients declined to participate. The age range of participants spanned from 18 to 79 years, with the largest group being between 30 and 39 years (n=10). The gender and age distribution are shown in Table 3. The survey also gathered data on the number and frequency of IT systems used for document management (Table 2). Fourteen participants did not use any systems, while others reported using between 1 and more than 3. The frequency of use varied, with 18 participants engaging with these systems at least once a week.

**Table 3.** Demographic data of participants for phase 3 (n=32).

Participant demographic	Value, n (%)
Sex	
Female	19 (59.4)
Male	13 (40.6)
Age (years)	
18‐29	1 (3.1)
30‐39	10 (31.3)
40‐49	5 (15.6)
50‐59	6 (18.8)
60‐69	8 (25)
70‐79	2 (6.3)
80‐89	0 (0)
IT^a^ systems used for document management	
0	14 (43.8)
1	12 (37.5)
2	5 (15.6)
3	0 (0)
>3	1 (3.1)
Frequency of IT system use for document management	
Never	14 (43.8)
Once a month	5 (15.6)
Several times a month	3 (9.4)
Once a week	4 (12.5)
Several times a week	3 (9.4)
Daily	3 (9.4)

a IT: Information Technology

### Evaluation Outcomes

All patients completed Tasks 1 and 4 without any assistance and correctly confirmed their connection to a health care facility (Task 1) and finding a nursing service (Task 4). Throughout Task 2, 6 patients (including both patients in the oldest age group [70-79 years]) needed assistance in carrying out the task. In Task 3, 1 patient received assistance in carrying out the task.

**Figure 6.**
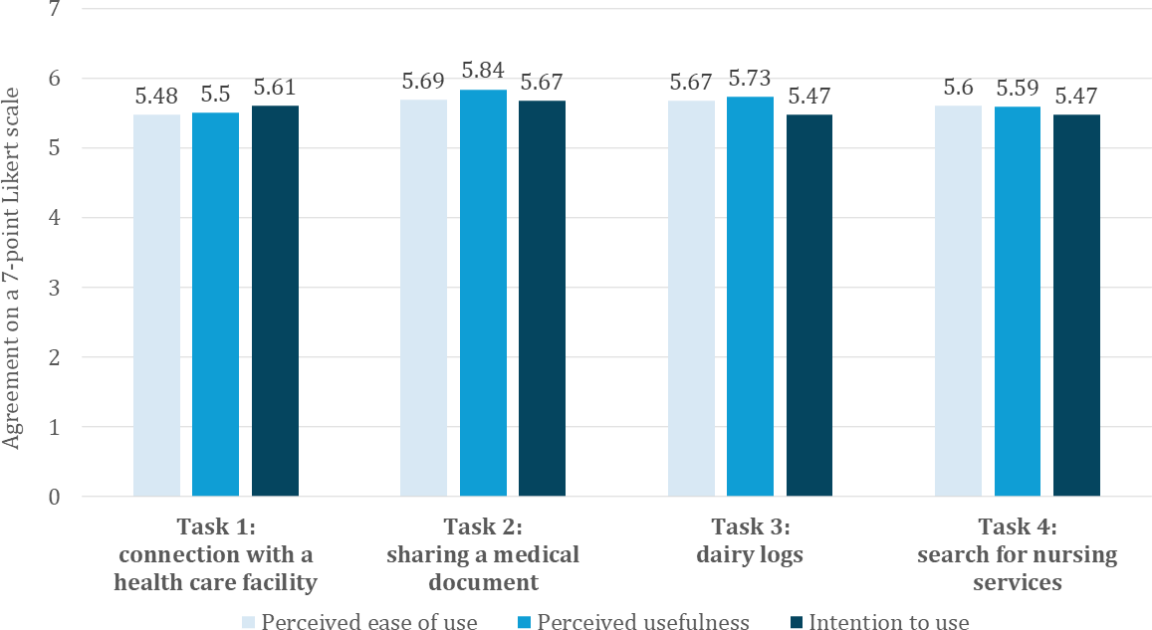
Perceived usefulness (PU), perceived ease of use (PEOU), and behavioral intention (BI) values and SDs for all 4 tasks of phase 3.

Table 4 provides an overview of the mean scores, SDs, and reliability measures (Cronbach α and Spearman-Brown coefficient) for the constructs PU, PEOU, and BI for each of the 4 tasks. A graphical comparison between the 3 constructs over the 4 tasks is shown in Figure 6. Overall, participants reported high levels of PU, PEOU, and BI for all tasks, with all reliability coefficients exceeding the recommended threshold of 0.70, displaying high internal consistency of the adapted scales [52].

**Table 4.** Technology acceptance model (TAM) construct measures for the blockchain (BC)-based health information exchange (HIE) app.

Construct	Task 1	Task 2	Task 3	Task 4
Perceived ease of use				
Value, mean (SD; 95% CI)	5.48 (1.42; 4.97-5.99)	5.69 (1.37; 5.19-6.18)	5.67 (1.33; 5.19-6.15)	5.6 (1.16; 5.18-6.02)
Cronbach 𝛼	0.94	0.93	0.96	0.95
Perceived usefulness				
Value, mean (SD; 95% CI)	5.5 (1.51; 4.96-6.04)	5.84 (1.28; 5.37-6.30)	5.73 (1.29; 5.26-6.19)	5.59 (1.14; 5.17-6.00)
Cronbach 𝛼	0.98	0.95	0.97	0.97
Intention to use				
Value, mean (SD; 95% CI)	5.61 (1.46; 5.08-6.14)	5.67 (1.6; 5.09-6.25)	5.47 (1.65; 4.87-6.06)	5.47 (1.28; 5.01-5.93)
Spearman-Brown coefficient	0.88	0.97	0.9	0.92

**Figure 7.**
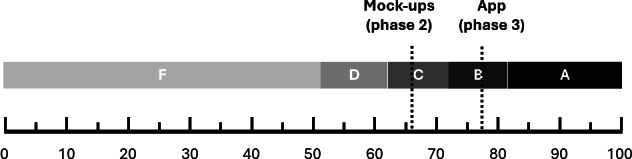
Grading of System Usability Scale (SUS) scores from phases 2 and 3.

The developed blockchain-based mobile app achieved a SUS score of 77.34, indicating that the usability was good and could be graded as “B+” [39,52]. This showed an improvement from the 66.50 (graded as “C”) reported in phase 2 (refer to Figure 7). It is noteworthy that older participants (ie, age groups 60‐69 and 70‐79 years) have a smaller SUS score of 64 (graded as “C–“) and hence only a satisfactory rating of the mobile app. In contrast, younger age groups (ie, 18‐59 years) have a SUS score of 83 (graded as “A”), indicating a high usability [51]. The results show that the BC-based mobile app received a higher SUS score of 84.12 (graded as “A”) among female participants. In contrast, male participants provided a rating of 67.43 (graded as “C”). We observed a minor difference in the SUS scores between participants who indicated that they would not use any document management system in general (74, graded as “B–“) and those who indicated using at least 1 such system (78.73, graded as “B+”). The SUS score of low frequencies of general document management system use was 75.61 (graded as “B”) and of high frequencies of general document management system use was 79.87 (graded as “A–”), showing only a minor difference.

For completeness, we report on a subgroup analysis using Aligned Rank Transform ANOVAs to examine the influence of gender, age, and number and frequency of IT systems used for document management on PEOU, PU, BI, and SUS (Multimedia Appendix 6). Aligned Rank Transform was used to align and rank the data to be able to apply ANOVA to our ordinal data [53]. Both analyses are exploratory and should be interpreted with caution given the limited sample size and the low statistical power.

### Influential Factors for User Perceptions

**Figure 8.**
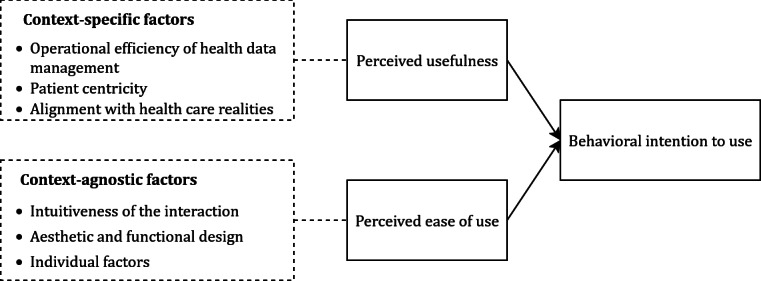
Mapping of factors influencing user perceptions to the technology acceptance model.

Our analysis of responses to the 3 open questions revealed 3 influential factors, each for PU and PEOU (refer to Table 5). The factors influencing PU reflect the specific contextual nuances of blockchain-based HIE, highlighting that users view a blockchain-based HIE application’s usefulness primarily through its ability to address specific challenges and requirements in this domain. In contrast, the factors shaping PEOU are context-agnostic, since they are not dependent on functionalities enabled by blockchain-based HIE. These align with broader user expectations for a general system’s usability and are straightforward to adopt across diverse settings (refer to Figure 8).

**Table 5.** Factors influencing user perceptions of blockchain (BC)-based health information exchange (HIE) applications.

Factors	Description	Exemplary quotes
Operational efficiency of health data management	The HIE^a^ application’s ability to organize and manage health-related information in a manner that reduces the time, effort, and resources required.	“Reduces bureaucratic overhead” “Everything is concentrated and easily accessible”
Patient-centeredness	The HIE application’s characteristic of placing patients at the center of its functionality by enabling them to manage their health data while ensuring transparency and privacy.	“I like the feature that I can set my release options personally” “Autonomous or not dependent on help from third parties”
Alignment with health care realities	The extent to which the HIE application reflects and supports the practical, real-world needs and workflows of health care settings.	“It would be nice if there was an appointment entry function” “The app should be available to everyone, including on mobile phones and via a browser.”
Intuitiveness of the interaction	The ability of the HIE application to provide a seamless and intuitive interaction experience.	“The search function for tutorials is helpful” “To approve a document, it is intuitive to first click on the document”
Aesthetic and functional design	The visual appeal and practical functionality of the HIE application.	“I would like to see a graphical representation” “The visual design is appealing”
Individual differences	Personal characteristics such as age, health status, and related experience that influence individuals’ attitudes toward the HIE application.	“I see that as very personality-dependent” “Older people may not be able to handle the many clicks.”

a HIE: health information exchange.

For PU, the following 3 factors were identified that can represent the key aspects users consider when evaluating the value and functionality of a blockchain-based HIE application.

### Operational Efficiency of Health Data Management

Our analysis identified the HIE application’s ability to organize and manage health-related information efficiently as a critical factor for its PU. This finding aligns with the highest PU score obtained for the document sharing task (Task 2), suggesting that participants particularly valued the application’s ability to facilitate data exchange between health care facilities and service providers. In particular, they highlighted the application’s strengths such as “faster document retrieval” and “easy sharing processes,” which streamline the exchange of health-related information. Furthermore, features that reduce administrative burdens, such as “simplified [processes to achieve] compliance with data policies” and “straightforward access to documents,” were highly valued for enhancing the efficiency of document retrieval. The organization of health data in one place also stood out. Participants appreciated the application’s “integrated view of all relevant data,” which provides a clear overview, ultimately supporting more effective health data management.

### Patient Centricity

Many participants attributed the positive PU of our HIE application to its user-centered advantages of empowering users to take ownership of their health data while protecting their privacy. This factor exerts an influence on participants’ perception of the application’s usefulness, as it allows them to manage their own data access and sharing settings, thereby fostering a sense of autonomy. Some users especially noted that “self-determined and self-organized document sharing” resulted in a more responsive system that better met their needs. Additionally, transparency in how user data is handled contributed to establishing trust in the system and enhanced users’ confidence in its utility. Participants expressed appreciation for “clear policies and measurements regarding data privacy,” with some indicating that “knowing their data was secure and handled responsibly” addressed their concerns related to privacy.

### Alignment With Healthcare Realities

Beyond the evaluated functions of the blockchain-based HIE application, participants emphasized the importance of aligning its capabilities with the health care setting’s structural and technological demands. Functions reflecting real-world healthcare scenarios, such as accessing medication plans and document sharing, were emphasized to enhance the application’s relevance and feasibility. This also helps explain the strong PU ratings for the document sharing task (Task 2). Moreover, participants suggested that additional features such as appointment scheduling, proof of insurance, and document search functions would further improve the PU. Although these were not tested in our survey, the participants considered them to be valuable for improving the usefulness of the application, as they can support people in their common activities of daily life. Furthermore, participants noted that the compatibility between the system and the existing infrastructure affects the PU of the application. Participants expressed that the application “should work seamlessly on different platforms or devices, including mobile phones and web browsers.” Moreover, maintaining “accessibility in locations with limited network connectivity” can reinforce the application’s usefulness in health care settings, as it facilitates uninterrupted service delivery and enables timely decision-making in critical situations when vital medical information is needed.

When reporting the PEOU, participants also identified 3 key factors that influence their perceptions:

### Intuitiveness of the Interaction

The ability of the HIE application to provide a seamless and intuitive interaction experience was reported in our survey as a critical factor influencing PEOU. Many participants stressed that the system should feel natural and require minimal learning, particularly for new users. For example, participants value features that facilitate convenient navigation, such as the “inclusion of a backspace function.” “Clear and consistent terminology” was also regarded as essential to “minimize [user] effort and frustration.” The positive feedback on interface clarity and navigation aligns with the marked improvement in SUS from phase 2 (66.5) to phase 3 (77.3), suggesting that these refinements in interaction design translated into perceptibly higher ease of use. In addition, participants identified built-in guidance, such as “a searchable help or tutorial feature,” as an important component to assist users in understanding the application’s functionality and resolving potential difficulties.

### Aesthetic and Functional Design

Several participants pointed out that the design of the HIE application plays an important role in PEOU. On the one hand, participants appreciated well-organized and visually appealing interfaces, as they made the application more attractive and easier to navigate. In particular, information displayed in structured timelines or graphical formats was considered helpful, such as “visually appealing curves” and “organized layouts by day, week, or month, [which] make [the information] easier to understand.” On the other hand, participants attributed high PEOU to customization options, including the ability to “enter free text” or “filter information by various criteria (eg, diagnoses and providers).” These options enable the application to better fulfill users’ individual needs, thereby fostering a more comfortable user experience and, ultimately, PEOU.

### Individual Differences

Personal characteristics emerge from our analysis as an influencing factor for PEOU. Given the health status of our participants, the first observation that caught our attention was that participants with serious health challenges reported that each new type of interaction felt less easy and more stressful. Besides, participants expressed concern about the application’s complexity for older users, noting that “too many steps or interactions could discourage [their] usage.” This observation aligns with concerns reported in phase 2 and the lower SUS scores reported by older participants in phase 3 (64 vs 83 for younger users), suggesting that PEOU was partly constrained by age-related differences. Furthermore, related experience and individual preferences further shaped perceptions. While some participants expressed discomfort or resistance due to limited familiarity with digital systems, 2 participants directly stated that their resistance stemmed solely from their personal aversion to digitization in general, not from the blockchain-based HIE application itself.

## Discussion

### Principal Findings

With this study, we provide empirical insights from the evaluation of a functional prototype of a patient-centric blockchain-based HIE mobile app in a clinical context. While prior studies largely examined hypothetical attitudes of blockchain-based HIE [3,27,28], our evaluation involves real patients undergoing medical treatment in a hospital, realistic usage scenarios, and realistic sample documents related to their care. By developing the blockchain-based HIE mobile app through a user-centered design approach in 3 phases, we incorporated patient feedback at every design stage to ensure that the prototype’s features and user interface aligned closely with patient needs. After iteratively refining the app, we conducted an evaluation with patients with cancer, assessing its usability, perceived value, and overall acceptance based on the SUS and TAM. While this evaluation context naturally limits the extent to which the results can be generalized to all patient groups or long-term use situations, it nevertheless provides a rigorous and informative test case for understanding how patients engage with blockchain-based HIE functionalities in practice. In the following, we discuss the main findings of our study.

First, our findings support the growing body of literature that suggests HIE as an auspicious use case for blockchain-based applications in health care [22]. Similar to prior studies focusing on users’ attitudes [3,15], the participants in our study expressed a positive attitude toward the blockchain-based functions that enable them to define, track, and revoke access to their health data. Although these findings stem from a specific patient population, the underlying interaction required by performing these functions is not disease-specific and reflects fundamental HIE tasks. This suggests that while generalization must be approached cautiously, the interactions we observe may hold relevance beyond the oncology context. The persistence of positive attitudes in our study reveals that a user-centered design process can effectively translate blockchain’s technical characteristics (eg, transparency and immutability) into comprehensible, patient-oriented functionalities. Furthermore, the high PEOU, PU, and BI ratings for the document-sharing function among all presented functionalities highlight the core need addressed by blockchain-based HIE applications, as these applications help to overcome the persistent challenges of managing patient data efficiently and securely in practice [22].

Second, by exploring the rationales behind reported user perceptions, we identified 6 influential factors for the 2 core TAM constructs (ie, PU and PEOU). While the 3 factors influencing PU reflect the specific contextual demands of HIE, the 3 factors shaping PEOU are context-agnostic. This strengthens prior findings, which highlight that PU depends on how well a system (eg, a HIE application) addresses the unique challenges and requirements of a particular context (eg, HIE) or task (eg, sharing a medical document) [54]. In contrast, PEOU reflects users’ perception of how easy and intuitive a system is to learn and operate. It is therefore influenced more by universal design factors that make the system user-friendly rather than its context of use [54].

Third, the age-related differences in perceived usability of our blockchain-based HIE resonate with a broad stream of eHealth and mobile health research indicating that older adults tend to experience higher usability barriers [55,56]. Although not significant, our results indicate a tendency of older adults to rate the overall usability of the mobile app lower than participants in younger age groups. In particular, among the surveyed patients, people from the age groups 60‐69 and 70‐79 years rated the mobile app’s usability as satisfactory only, while the younger age groups rated the usability of the mobile app as good. This concurs with previous research on digital health equity issues that emphasizes that older adults are generally less experienced with mobile apps, leading to a lower usability [55,56]. Nevertheless, this also highlights that the development of digital health apps must account for the higher needs of older adults for simplified navigation and intuitive interactions to overcome age-related barriers [57]. Addressing these barriers prevents digital health equity gaps and ensures that they can use and benefit from HIE mobile apps. Toward that end, we deem user-centered design approaches especially useful since, in our study, for example, we were able to improve the usability ratings in the older age groups through multiple iterations.

### Implications

Our study has several implications for research and practice. In terms of research, we add to the growing body of research on patient perceptions of blockchain-based HIE by evaluating the perceptions of cancer patients concerning interactions with a blockchain-based HIE mobile app in a clinical context. Furthermore, we combine quantitative survey results (ie, the SUS scores) with the in-depth insights from a qualitative survey of patients to expand our understanding of patient perceptions of blockchain-based HIE in a naturalistic setting. In particular, the results of the qualitative survey paint a more nuanced picture of context-specific and context-agnostic factors that are crucial for the broad acceptance of blockchain-based HIE. In addition, our study also highlights the value of user-centered design studies in the context of developing and evaluating blockchain-based applications for health care. Although blockchain is primarily a back-end technology, the decentralization of IT systems associated with the use of BC also has an indirect impact on the user experience. Against this backdrop, user-centered design studies can help to better understand user requirements and generate design knowledge for blockchain-based HIE applications.

From a practitioner perspective, our study yields implications particularly for healthcare policymakers and developers of blockchain-based HIE. For healthcare policymakers, the results of our evaluation highlight the potential of blockchain-based HIE to alleviate deficiencies in the current care system, such as lost documents, a time-consuming process to collect and share relevant medical documents, or incomplete health data for patients with cancer. Although many of these challenges are being addressed through the implementation of national health infrastructures, such as electronic health records (eg, [9]), patients remain hesitant to fully engage with these systems due to concerns about privacy, control, or usability, even in contexts where national systems automatically enroll patients unless they actively opt out [58]. Our results indicate that blockchain-based HIE solutions can address these concerns by fostering patient centricity and privacy. Therefore, our results highlight broader dimensions beyond basic usability measures, including patient empowerment and transparency, which are critical for implementing and integrating new technologies into patient-related health care workflows [59]. Participants in our study valued the ability to manage data access and sharing, which strengthened their sense of autonomy and increased the PU of our suggested blockchain-based HIE system. Such systems may be particularly beneficial in less connected areas, such as rural health care settings, where digital infrastructure and interoperability are limited. Pilot projects and model regions in these contexts could provide valuable insights for further scaling and optimizing these systems. Furthermore, for developers of BC-based HIE applications, our study provides a promising design for a BC-based HIE mobile app. When implementing blockchain-based HIE in practice, the identified factors related to the PU and the PEOU should be considered, since both will help ensure that patients use and accept a blockchain-based HIE mobile app. To ensure and improve PU, blockchain-based HIE mobile apps should focus on building functionalities that put all patient data into one overview to reduce time and effort in data sharing. Moreover, medical documents should be shareable individually, and patients should be able to effortlessly establish a connection with health care providers (eg, via a QR code). For ensuring and improving the PEOU, future developments should provide an intuitive design and minimize the number of clicks it takes to achieve a task (eg, share a medical document).

### Limitations and Future Research

Our study is not without limitations. First, during the evaluation, patients could ask questions if they felt stuck while interacting with the mock-ups in phase 2 and the mobile app in phase 3. While we tried to provide minimal guidance across all surveyed patients, some asked for more assistance than others. This might have skewed the results in favor of a higher SUS, PEOU, PU, and BI and limited the comparability of patient assessments. Even though participants rarely requested guidance during tasks, the mere presence of help at hand could have led to participants feeling more confident in their interactions, thereby increasing their overall perceptions of the mobile app. Future research could extend our study by examining user perceptions without providing a contact person for assistance. Second, in our collection of patients’ responses, we focused on patients with cancer in a single major university hospital in Germany because their treatment requires exchanging patients’ health data across multiple health care providers and because their physical and emotional state demands a high usability of the mobile app. This focus might reduce the applicability of our results for other patient groups. Other patient groups might prefer sharing different documents (eg, vaccination record) or might even attach less importance to the concept of blockchain-based HIE since their situation does not demand frequent sharing of documents. Also, patient perceptions might be different when they have positively or negatively experienced the document management process in other hospitals. Future research could hence extend the evaluation of blockchain-based mobile apps to other patient groups and other hospitals. Third, we tried to incorporate a broad range of views in the development of the app through quantitative measures and qualitative insights. However, the convenience sampling used and the limited sample sizes in each phase of the evaluation might have introduced a selection bias and limited the generalizability of our results to a broader population. Future research should validate our findings in a large, balanced sample. This will be especially useful to draw further conclusions about the differences in participants’ characteristics (eg, age) influencing patient perceptions (eg, PEOU, usefulness, or acceptance). Finally, we evaluated patient interactions with the blockchain-based HIE mobile app only at 1 point in time. Future research could extend this study by evaluating the interactions with a blockchain-based HIE mobile app over time. Such a longitudinal study could help uncover insights into actual patient sharing behavior and determine whether patients would use a mobile app over a longer timeframe.

### Conclusion

With our study, we provide an exemplary case of a blockchain-based HIE mobile app developed through a 3-phase user-centered design approach incorporating on-site patients from a major university hospital. Our evaluation shows that patients in our study would likely use a blockchain-based HIE mobile app to manage their health data, since the app enables them to decide which health care provider or individuals can access their data.

## Supplementary material

10.2196/78849 Multimedia Appendix 1 Phase 1 questionnaire and frequency analysis.

10.2196/78849 Multimedia Appendix 2 Screenshots of the blockchain (BC)-based HIE mock-ups.

10.2196/78849 Multimedia Appendix 3 Screenshots of the blockchain (BC)-based HIE mobile app.

10.2196/78849 Multimedia Appendix 4 Phase 3 questionnaire.

10.2196/78849 Multimedia Appendix 5 Phase 3 technology acceptance model (TAM) and System Usability Scale (SUS) frequency analysis.

10.2196/78849 Multimedia Appendix 6 Phase 3 ANOVA results for technology acceptance model (TAM) and System Usability Scale (SUS).

## Abbreviations

BI: behavioral intention
HIE: health information exchange
PEOU: perceived ease of use
PU: perceived usefulness
SUS: System Usability Scale
TAM: technology acceptance model
