# Supplementary material for: Patient Perceptions of Blockchain-Based Health Information Exchange: User-Centered Design Study
Source: J Med Internet Res. 2026 Mar 11;28:e78849. doi: 10.2196/78849 (PMC13000691; doi:10.2196/78849)
Supplement: Multimedia Appendix 1 [file jmir-v28-e78849-s001.docx]

**Supplementary Table.** Phase 1 Patient survey responses.

| **Variables** | **Participants (n=60)** |
| --- | --- |
|  |  |
| **Have you ever experienced medical documents being lost, forgotten, or mixed up? [I myself have lost documents.]** |  |
| Yes | 7 (11.7%) |
| No | 53 (88.3%) |
| **Have you ever experienced medical documents being lost, forgotten, or mixed up? [Medical staff lost documents.]** |  |
| Yes | 6 (10.0%) |
| No | 54 (90.0%) |
| **Have you ever experienced medical documents being lost, forgotten, or mixed up? [Medical staff did not provide me with documents on time.]** |  |
| Yes | 14 (76.7%) |
| No | 46 (23.3%) |
| **Have you ever experienced medical documents being lost, forgotten, or mixed up? [I forgot to bring documents.]** |  |
| Yes | 16 (26.7%) |
| No | 44 (73.3%) |
| **Have you ever experienced medical documents being lost, forgotten, or mixed up? [Medical documents were not available to me during treatment.]** |  |
| Yes | 16 (26.7%) |
| No | 44 (73.3%) |
| **Have you ever experienced medical documents being lost, forgotten, or mixed up? [It never happened that medical documents were lost, forgotten, or mixed up.]** |  |
| Yes | 30 (50.0%) |
| No | 30 (50.0%) |
| **Who has an overview of your health documents? [I have a complete overview of my health documents]** |  |
| Strongly agree | 12 (20.0%) |
| Agree | 21 (35.0%) |
| Somewhat agree | 13 (21.7%) |
| Somewhat disagree | 9 (15.0%) |
| Disagree | 4 (6.7%) |
| Strongly Disagree | 1 (1.7%) |
| **Who has an overview of your health documents? [My general practitioner has a complete overview of my health documents]** |  |
| Strongly agree | 3 (5.0%) |
| Agree | 8 (13.3%) |
| Somewhat agree | 13 (21.7%) |
| Somewhat disagree | 20 (33.3%) |
| Disagree | 12 (20.0%) |
| Strongly Disagree | 4 (6.7%) |
| **Who has an overview of your health documents? [My relatives have a complete overview of my health documents]** |  |
| Strongly agree | 3 (5.0%) |
| Agree | 3 (5.0%) |
| Somewhat agree | 4 (6.7%) |
| Somewhat disagree | 10 (16.7%) |
| Disagree | 20 (33.3%) |
| Strongly Disagree | 20 (33.3%) |
|  |  |
| **Would direct access to all your health documents via smartphone, tablet, or computer be helpful? [Access via smartphone would be helpful.]** |  |
| Strongly agree | 33 (55.0%) |
| Agree | 11 (18.3%) |
| Somewhat agree | 6 (10.0%) |
| Somewhat disagree | 7 (11.7%) |
| Disagree | 1 (1.7%) |
| Strongly Disagree | 2 (3.3%) |
| **Would direct access to all your health documents via smartphone, tablet, or computer be helpful? [Access via tablet would be helpful.]** |  |
| Strongly agree | 26 (43.3%) |
| Agree | 14 (23.3%) |
| Somewhat agree | 4 (6.7%) |
| Somewhat disagree | 6 (10.0%) |
| Disagree | 5 (8.3%) |
| Strongly Disagree | 5 (8.3%) |
| **Would direct access to all your health documents via smartphone, tablet, or computer be helpful? [Access via computer would be helpful.]** |  |
| Strongly agree | 39 (65.0%) |
| Agree | 13 (21.7%) |
| Somewhat agree | 5 (8.3%) |
| Somewhat disagree | 1 (1.7%) |
| Disagree | 1 (1.7%) |
| Strongly Disagree | 1 (1.7%) |
| **Evaluation of our solution – To what extent do you agree with the following statements about our new application? [I think I would like to use this application frequently.]** |  |
| Strongly agree | 23 (38.3%) |
| Agree | 24 (40.0%) |
| Neutral | 10 (16.7%) |
| Disagree | 2 (3.3%) |
| Strongly Disagree | 1 (1.7%) |
| **The following documents would be relevant for me in the application: [Medical findings, diagnoses, and surgical reports]** |  |
| Strongly agree | 40 (66.7%) |
| Agree | 17 (28.3%) |
| Somewhat agree | 2 (3.3%) |
| Somewhat disagree | 0 (0.0%) |
| Disagree | 0 (0.0%) |
| Strongly Disagree | 1 (1.7 %) |
| **The following documents would be relevant for me in the application: [Laboratory results, such as blood test results]** |  |
| Strongly agree | 41 (68.3%) |
| Agree | 17 (28.3%) |
| Somewhat agree | 1 (1.7%) |
| Somewhat disagree | 0 (0.0%) |
| Disagree | 0 (0.0%) |
| Strongly Disagree | 1 (1.7 %) |
| **The following documents would be relevant for me in the application: [Medication plan]** |  |
| Strongly agree | 36 (60.0%) |
| Agree | 15 (25.0%) |
| Somewhat agree | 4 (6.7%) |
| Somewhat disagree | 1 (1.7%) |
| Disagree | 3 (5.0%) |
| Strongly Disagree | 1 (1.7 %) |
| **The following documents would be relevant for me in the application: [Imaging (CT, MRI, etc.)]** |  |
| Strongly agree | 35 (58.3%) |
| Agree | 17 (28.3%) |
| Somewhat agree | 5 (8.3%) |
| Somewhat disagree | 1 (1.7%) |
| Disagree | 0 (0.0%) |
| Strongly Disagree | 2 (3.3 %) |
| **The following documents would be relevant for me in the application: [Contracts and treatment costs]** |  |
| Strongly agree | 16 (26.7%) |
| Agree | 19 (31.7%) |
| Somewhat agree | 14 (23.3%) |
| Somewhat disagree | 6 (10.0%) |
| Disagree | 3 (5.0%) |
| Strongly Disagree | 2 (3.3%) |

| **The following documents would be relevant for me in the application: [Consent forms for medical procedures and treatments]** |  |
| --- | --- |
| Strongly agree | 21 (35.0%) |
| Agree | 15 (25.0%) |
| Somewhat agree | 10 (16.7%) |
| Somewhat disagree | 10 (16.7%) |
| Disagree | 2 (3.3%) |
| Strongly Disagree | 2 (3.3%) |
| **The following documents would be relevant for me in the application: [Vital signs (blood pressure, heart rate, temperature)]** |  |
| Strongly agree | 22 (36.7%) |
| Agree | 19 (31.7%) |
| Somewhat agree | 12 (20.0%) |
| Somewhat disagree | 5 (8.3%) |
| Disagree | 1 (1.7%) |
| Strongly Disagree | 1 (1.7%) |
| **The following documents would be relevant for me in the application: [Advance healthcare directive and power of attorney for healthcare]** |  |
| Strongly agree | 32 (53.3%) |
| Agree | 14 (23.3%) |
| Somewhat agree | 8 (13.3%) |
| Somewhat disagree | 2 (3.3%) |
| Disagree | 2 (3.3%) |
| Strongly Disagree | 2 (3.3%) |
| **The following documents would be relevant for me in the application: [Vaccination record]** |  |
| Strongly agree | 36 (60.0%) |
| Agree | 17 (28.3%) |
| Somewhat agree | 4 (6.7%) |
| Somewhat disagree | 1 (1.7%) |
| Disagree | 1 (1.7%) |
| Strongly Disagree | 1 (1.7%) |
| **The following documents would be relevant for me in the application: [Prescriptions and medical orders]** |  |
| Strongly agree | 29 (48.3%) |
| Agree | 21 (35.0%) |
| Somewhat agree | 7 (11.7%) |
| Somewhat disagree | 2 (3.3%) |
| Disagree | 0 (0.0%) |
| Strongly Disagree | 1 (1.7%) |

| **The following documents would be relevant for me in the application: [Applications and certificates (e.g., sick leave certificates and rehabilitation forms)]** |  |
| --- | --- |
| Strongly agree | 29 (48.3%) |
| Agree | 18 (30.0%) |
| Somewhat agree | 4 (6.7%) |
| Somewhat disagree | 6 (10.0%) |
| Disagree | 2 (3.3%) |
| Strongly Disagree | 1 (1.7%) |
| **I would share the following documents with others via the application: [Medical findings, diagnoses, and surgical reports]** |  |
| Yes | 53 (88.3%) |
| No | 7 (11.7%) |
| **I would share the following documents with others via the application: [Laboratory results, such as blood test results]** |  |
| Yes | 52 (86.7%) |
| No | 8 (13.3%) |
| **I would share the following documents with others via the application: [Medication plan]** |  |
| Yes | 51 (85.0%) |
| No | 9 (15.0%) |
| **I would share the following documents with others via the application: [Imaging (CT, MRI, etc.)]** |  |
| Yes | 52 (86.7%) |
| No | 8 (13.3%) |
| **I would share the following documents with others via the application: [Contracts and treatment costs]** |  |
| Yes | 20 (33.3%) |
| No | 40 (66.7%) |
| **I would share the following documents with others via the application: [Consent forms for medical procedures or treatments]** |  |
| Yes | 30 (50.0%) |
| No | 30 (50.0%) |
| **I would share the following documents with others via the application: [Advance healthcare directive and power of attorney for healthcare]** |  |
| Yes | 40 (66.7%) |
| No | 20 (33.3%) |
| **I would share the following documents with others via the application: [Vaccination record]** |  |
| Yes | 46 (76.7%) |
| No | 14 (23.3%) |
| **I would share the following documents with others via the application: [Prescriptions and medical orders]** |  |
| Yes | 31 (51.7%) |
| No | 29 (48.3%) |
| **I would share the following documents with others via the application: [Applications and certificates (e.g., sick leave certificates and rehabilitation forms)]** |  |
| Yes | 31 (51.7%) |
| No | 29 (48.3%) |
| **The following documents would be relevant for me in the application: [Prescriptions and medical orders]** |  |
| Strongly agree | 29 (48.3%) |
| Agree | 21 (35.0%) |
| Somewhat agree | 7 (11.7%) |
| Somewhat disagree | 2 (3.3%) |
| Disagree | 0 (0.0%) |
| Strongly Disagree | 1 (1.7%) |
| **In our application, you can freely choose which individuals or institutions are granted specific rights. How do you evaluate this feature? [I want to individually decide who can edit certain documents.]** |  |
| Strongly agree | 37 (61.7%) |
| Agree | 11 (18.3%) |
| Somewhat agree | 8 (13.3%) |
| Somewhat disagree | 3 (5.0%) |
| Disagree | 0 (0.0%) |
| Strongly Disagree | 1 (1.7%) |
| **In our application, you can freely choose which individuals or institutions are granted specific rights. How do you evaluate this feature? [I want to individually decide who can delete certain documents.]** |  |
| Strongly agree | 45 (75.0%) |
| Agree | 11 (18.3%) |
| Somewhat agree | 2 (3.3%) |
| Somewhat disagree | 1 (1.7%) |
| Disagree | 0 (0.0%) |
| Strongly Disagree | 1 (1.7%) |
| **Our application offers the ability to track who accessed your shared documents and data and when (access history). How do you evaluate this feature? [This function is important to me.]** |  |
| Strongly agree | 35 (58.3%) |
| Agree | 17 (28.3%) |
| Somewhat agree | 3 (5.0%) |
| Somewhat disagree | 3 (5.0%) |
| Disagree | 2 (3.3%) |
| Strongly Disagree | 0 (0.0%) |
