# Supplementary material for: Patient Perceptions of Blockchain-Based Health Information Exchange: User-Centered Design Study
Source: J Med Internet Res. 2026 Mar 11;28:e78849. doi: 10.2196/78849 (PMC13000691; doi:10.2196/78849)

The mockups show a profile for a patient with (1) the access and sharing history for patient documents, (2) an overview of a medication plan, (3) a medication calendar, (4) an overview of all patient files, and (5) the security and confidentiality options for patient file sharing.


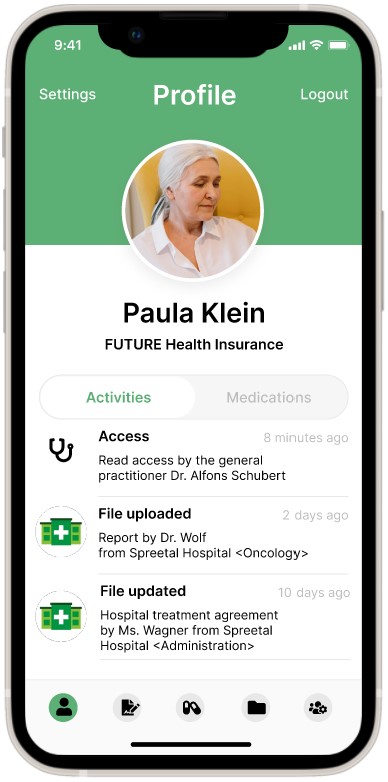

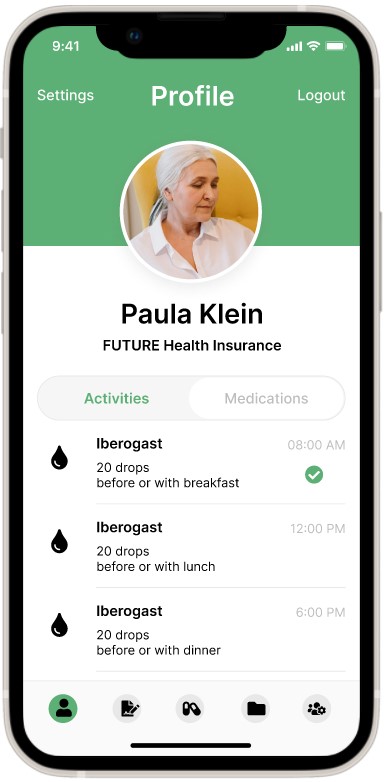

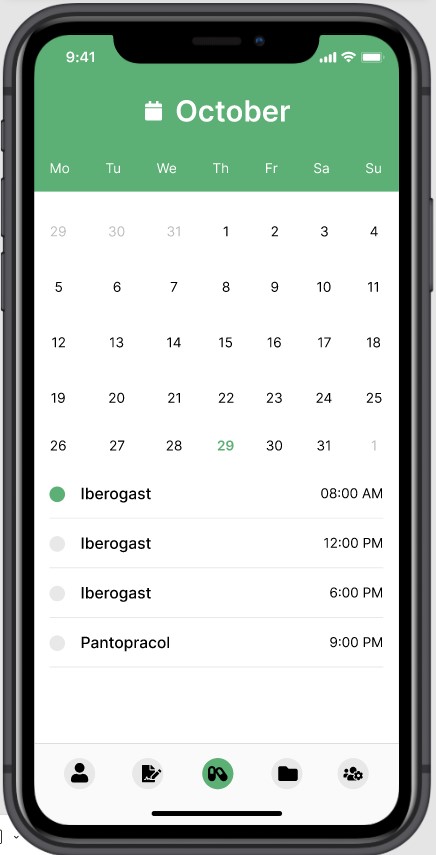

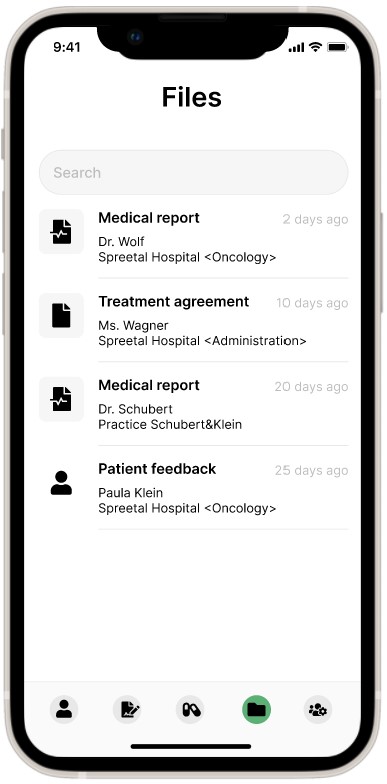

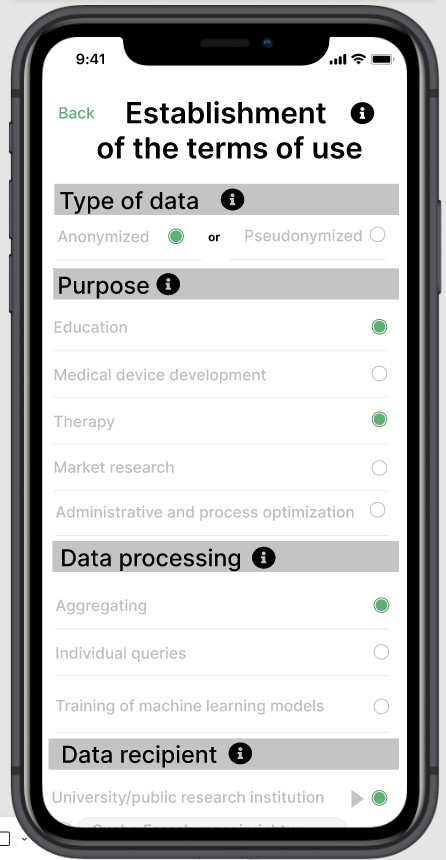

Supplement: Multimedia Appendix 2 [file jmir-v28-e78849-s002.docx]
