# Supplementary material for: Patient Perceptions of Blockchain-Based Health Information Exchange: User-Centered Design Study
Source: J Med Internet Res. 2026 Mar 11;28:e78849. doi: 10.2196/78849 (PMC13000691; doi:10.2196/78849)

The screenshots of the BC-based HIE application show (1) a profile for a patient, (2) a calendar for diary entries, (3) the sliders of the patient diary, (4) an overview of all patient documents, and (5) the selection of healthcare facilities and service providers for sharing patient documents, (6) an overview of how long a patient document shall be shared, and (7) an overview of connected healthcare facilities and service providers.


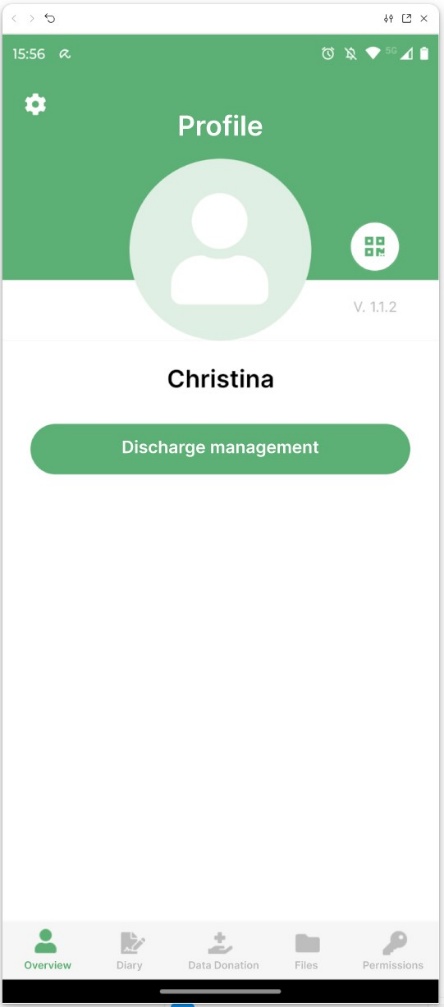

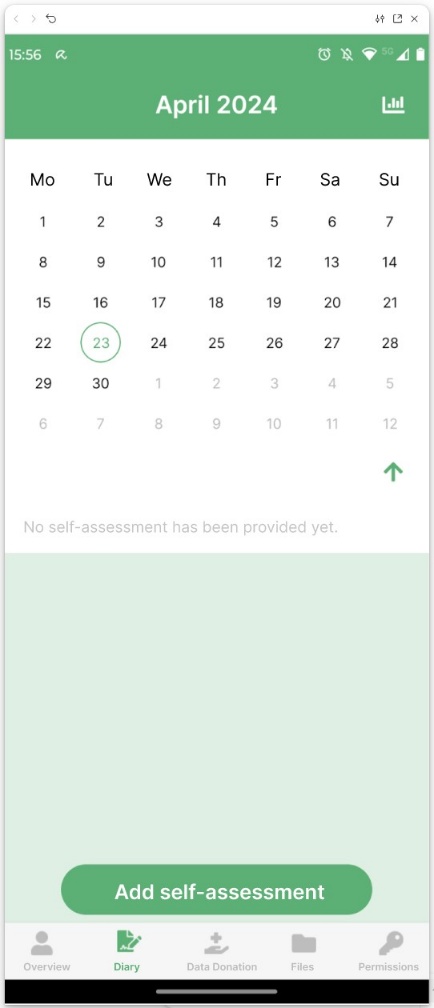

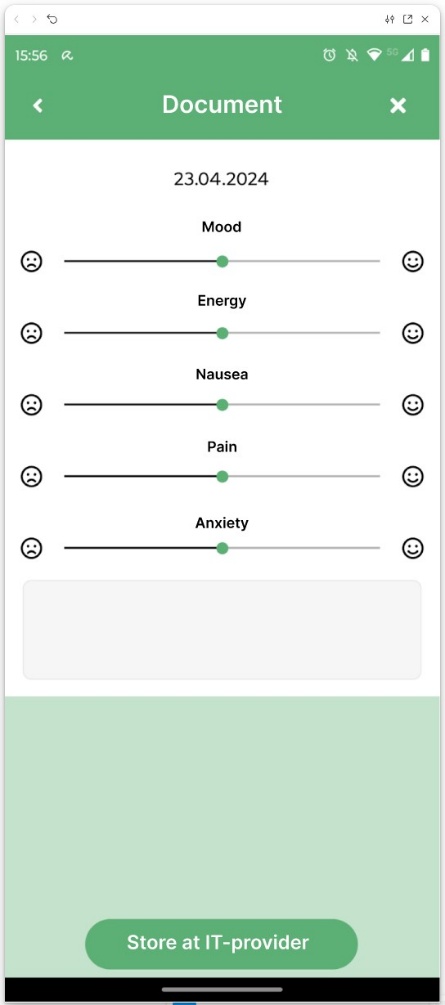

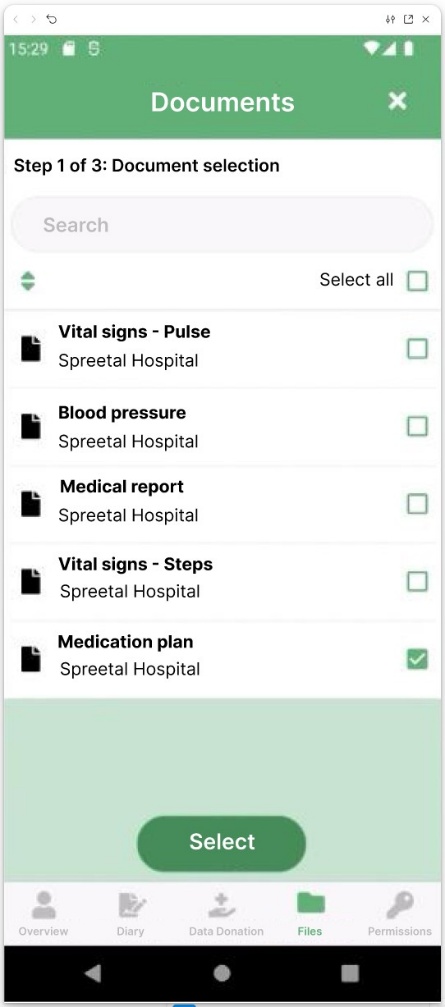

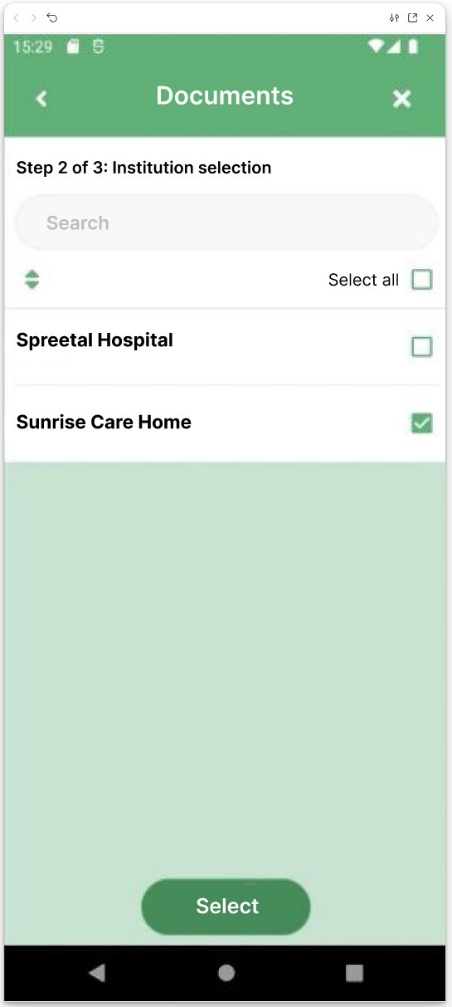

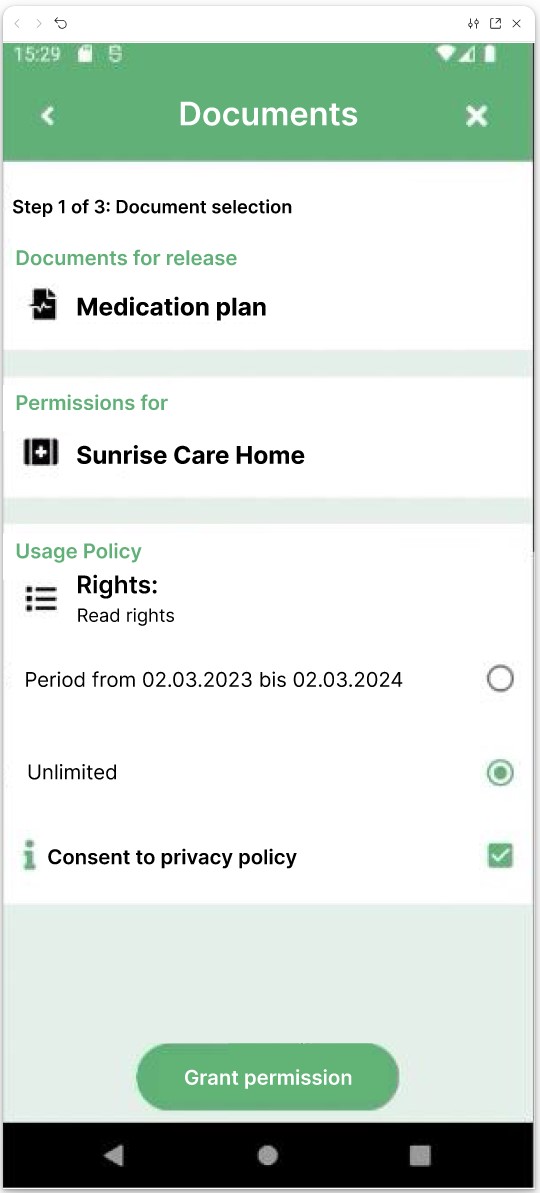

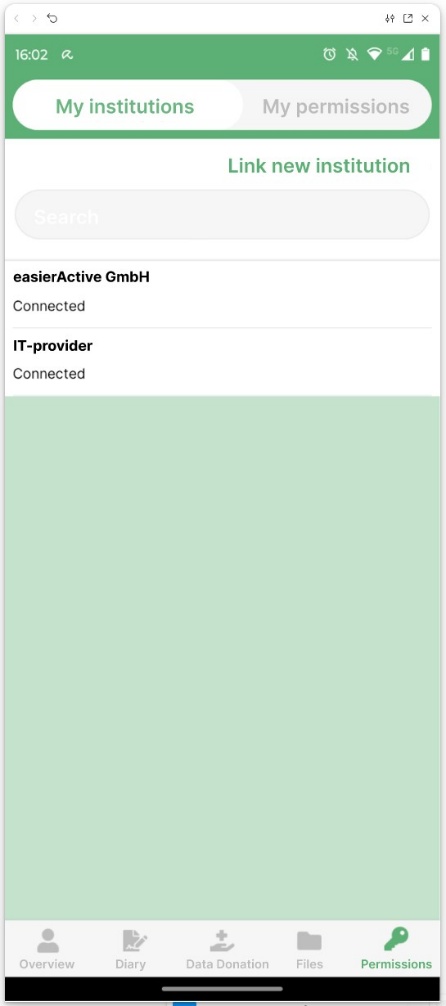

Supplement: Multimedia Appendix 3 [file jmir-v28-e78849-s003.docx]
