# Supplementary material for: Patient Perceptions of Blockchain-Based Health Information Exchange: User-Centered Design Study
Source: J Med Internet Res. 2026 Mar 11;28:e78849. doi: 10.2196/78849 (PMC13000691; doi:10.2196/78849)
Supplement: Multimedia Appendix 4 [file jmir-v28-e78849-s004.docx]

**Before you start the first task, please briefly answer the following questions about yourself:**

1. **Gender:**  Female  Male  Diverse  Prefer not to say
2. **Age range:**  18-29  30-39  40-49  50-59  60-69  70-79  80-89  >= 90  Prefer not to say
3. **Highest level of education:**

☐ No school diploma
☐ Secondary school diploma (lower level)
☐ Secondary school diploma (intermediate level)
☐ Vocational training/Skilled worker, Master craftsman
☐ High school diploma (Abitur)
☐ University degree (Bachelor’s)
☐ University degree (Master’s / Diploma)
☐ Doctorate (Dr. / PhD)
☐ Other, please specify:
☐ Prefer not to say

1. **Industry/Sector:**

☐ Healthcare
☐ Media & Entertainment
☐ Telecommunications & IT
☐ Other, please specify:
☐ Prefer not to say

1. **Number of IT systems used for document management (e.g., health insurance app, Google Drive, Dropbox, OneDrive):**

0

1

2

3

> 3

1. **Frequency of using IT systems for document management (e.g., health insurance app, Google Drive, Dropbox, OneDrive:**

☐ Daily
☐ Several times a week
☐ Once a week
☐ Several times a month
☐ Once a month
☐ Less frequently
☐ Never

**Introduction to the BloG³-System**

The BloG³ system is a digital platform that provides patients and healthcare providers (e.g., hospitals, general practitioners, etc.) with transparent access to personal health data at any time – despite the decentralized storage of data by the providers. Additionally, optional data from wearable health devices (e.g., smartwatches) can be integrated. Through the BloG³ system, patients can retrieve their health data from various healthcare providers that are connected to the platform. Furthermore, patients can share their own health data with providers via the BloG³ system. Sharing and accessing one's distributed health data in this way can enable personalized medical care, leading to faster and improved treatment. In summary, the BloG³ system can establish a decentralized data and access management approach while consolidating scattered health records into an individual health profile.

**BLOCK A**

**Please indicate your level of agreement of each of these statements in regard to your overall experiences of the connection with institutions.**

|  | ***Strongly disagree*** | ***Agree*** | ***Rather agree*** | ***Neu-tral*** | ***Rather Agree*** | ***Agree*** | ***Strongly Agree*** |
| --- | --- | --- | --- | --- | --- | --- | --- |
| 1. Using the BloG3 app improves my performance in completing such a task. | ① | ② | ③ | ④ | ⑤ | ⑥ | ⑦ |
| 1. Using the BloG3 app increases my productivity in completing such a task. | ① | ② | ③ | ④ | ⑤ | ⑥ | ⑦ |
| 1. Using the BloG3 app increases my effectiveness in completing such a task. | ① | ② | ③ | ④ | ⑤ | ⑥ | ⑦ |
| 1. I find the BloG3 app useful for completing such a task. | ① | ② | ③ | ④ | ⑤ | ⑥ | ⑦ |
| 1. Using the BloG3 app is clear and understandable when completing such a task. | ① | ② | ③ | ④ | ⑤ | ⑥ | ⑦ |
| 1. Using the BloG3 app does not require much mental effort when completing such a task. | ① | ② | ③ | ④ | ⑤ | ⑥ | ⑦ |
| 1. I find the BloG3 app easy to use when completing such a task. | ① | ② | ③ | ④ | ⑤ | ⑥ | ⑦ |
| 1. I find it easy to get the BloG3 app to do what I want when completing such task. | ① | ② | ③ | ④ | ⑤ | ⑥ | ⑦ |
| 1. Assuming I had access to the BloG3 app, I intend to use it. | ① | ② | ③ | ④ | ⑤ | ⑥ | ⑦ |
| 1. Provided I had access to the BloG3 app, I would predict that I would use it. | ① | ② | ③ | ④ | ⑤ | ⑥ | ⑦ |

**Block B**

**Please indicate your level of agreement with each of these statements in regard to your overall experiences of the document-sharing function.**

|  | ***Strongly disagree*** | ***Agree*** | ***Rather agree*** | ***Neu-tral*** | ***Rather Agree*** | ***Agree*** | ***Strongly Agree*** |
| --- | --- | --- | --- | --- | --- | --- | --- |
| 1. Using the BloG3 app improves my performance in completing such a task. | ① | ② | ③ | ④ | ⑤ | ⑥ | ⑦ |
| 1. Using the BloG3 app increases my productivity in completing such a task. | ① | ② | ③ | ④ | ⑤ | ⑥ | ⑦ |
| 1. Using the BloG3 app increases my effectiveness in completing such a task. | ① | ② | ③ | ④ | ⑤ | ⑥ | ⑦ |
| 1. I find the BloG3 app useful for completing such a task. | ① | ② | ③ | ④ | ⑤ | ⑥ | ⑦ |
| 1. Using the BloG3 app is clear and understandable when completing such a task. | ① | ② | ③ | ④ | ⑤ | ⑥ | ⑦ |
| 1. Using the BloG3 app does not require much mental effort when completing such a task. | ① | ② | ③ | ④ | ⑤ | ⑥ | ⑦ |
| 1. I find the BloG3 app easy to use when completing such a task. | ① | ② | ③ | ④ | ⑤ | ⑥ | ⑦ |
| 1. I find it easy to get the BloG3 app to do what I want when completing such task. | ① | ② | ③ | ④ | ⑤ | ⑥ | ⑦ |
| 1. Assuming I had access to the BloG3 app, I intend to use it. | ① | ② | ③ | ④ | ⑤ | ⑥ | ⑦ |
| 1. Provided I had access to the BloG3 app, I would predict that I would use it. | ① | ② | ③ | ④ | ⑤ | ⑥ | ⑦ |

**Block C**

**Please indicate your level of agreement with each of these statements in regard to your overall experience of the health diary function.**

|  | ***Strongly disagree*** | ***Agree*** | ***Rather agree*** | ***Neu-tral*** | ***Rather Agree*** | ***Agree*** | ***Strongly Agree*** |
| --- | --- | --- | --- | --- | --- | --- | --- |
| 1. Using the BloG3 app improves my performance in completing such a task. | ① | ② | ③ | ④ | ⑤ | ⑥ | ⑦ |
| 1. Using the BloG3 app increases my productivity in completing such a task. | ① | ② | ③ | ④ | ⑤ | ⑥ | ⑦ |
| 1. Using the BloG3 app increases my effectiveness in completing such a task. | ① | ② | ③ | ④ | ⑤ | ⑥ | ⑦ |
| 1. I find the BloG3 app useful for completing such a task. | ① | ② | ③ | ④ | ⑤ | ⑥ | ⑦ |
| 1. Using the BloG3 app is clear and understandable when completing such a task. | ① | ② | ③ | ④ | ⑤ | ⑥ | ⑦ |
| 1. Using the BloG3 app does not require much mental effort when completing such a task. | ① | ② | ③ | ④ | ⑤ | ⑥ | ⑦ |
| 1. I find the BloG3 app easy to use when completing such a task. | ① | ② | ③ | ④ | ⑤ | ⑥ | ⑦ |
| 1. I find it easy to get the BloG3 app to do what I want when completing such task. | ① | ② | ③ | ④ | ⑤ | ⑥ | ⑦ |
| 1. Assuming I had access to the BloG3 app, I intend to use it. | ① | ② | ③ | ④ | ⑤ | ⑥ | ⑦ |
| 1. Provided I had access to the BloG3 app, I would predict that I would use it. | ① | ② | ③ | ④ | ⑤ | ⑥ | ⑦ |

**Block D**

**Please indicate your level of agreement with each of these statements regarding your overall experience of the care service search function.**

|  | ***Strongly disagree*** | ***Agree*** | ***Rather agree*** | ***Neu-tral*** | ***Rather Agree*** | ***Agree*** | ***Strongly Agree*** |
| --- | --- | --- | --- | --- | --- | --- | --- |
| 1. Using the BloG3 app improves my performance in completing such a task. | ① | ② | ③ | ④ | ⑤ | ⑥ | ⑦ |
| 1. Using the BloG3 app increases my productivity in completing such a task. | ① | ② | ③ | ④ | ⑤ | ⑥ | ⑦ |
| 1. Using the BloG3 app increases my effectiveness in completing such a task. | ① | ② | ③ | ④ | ⑤ | ⑥ | ⑦ |
| 1. I find the BloG3 app useful for completing such a task. | ① | ② | ③ | ④ | ⑤ | ⑥ | ⑦ |
| 1. Using the BloG3 app is clear and understandable when completing such a task. | ① | ② | ③ | ④ | ⑤ | ⑥ | ⑦ |
| 1. Using the BloG3 app does not require much mental effort when completing such a task. | ① | ② | ③ | ④ | ⑤ | ⑥ | ⑦ |
| 1. I find the BloG3 app easy to use when completing such a task. | ① | ② | ③ | ④ | ⑤ | ⑥ | ⑦ |
| 1. I find it easy to get the BloG3 app to do what I want when completing such task. | ① | ② | ③ | ④ | ⑤ | ⑥ | ⑦ |
| 1. Assuming I had access to the BloG3 app, I intend to use it. | ① | ② | ③ | ④ | ⑤ | ⑥ | ⑦ |
| 1. Provided I had access to the BloG3 app, I would predict that I would use it. | ① | ② | ③ | ④ | ⑤ | ⑥ | ⑦ |

**Block E**

**Please indicate your level of agreement with each of these statements in regard to the BloG^3^-App in general.**

|  | ***Strongly Agree*** | ***Agree*** | ***Neutral*** | ***Disagree*** | ***Strongly Disagree*** |
| --- | --- | --- | --- | --- | --- |
| 1. I think I would like to use the BloG3 app frequently. | ① | ② | ③ | ④ | ⑤ |
| 1. I found the BloG3 app unnecessarily complex. | ① | ② | ③ | ④ | ⑤ |
| 1. I thought the BloG3 app was easy to use. | ① | ② | ③ | ④ | ⑤ |
| 1. I think I need the help of a tech-savvy person to use the BloG3 app. | ① | ② | ③ | ④ | ⑤ |
| 1. I found that the various functions were well integrated into the BloG3 app. | ① | ② | ③ | ④ | ⑤ |
| 1. I thought the BloG3 app was not consistent enough. | ① | ② | ③ | ④ | ⑤ |
| 1. I would imagine that most people would learn to use the BloG3 app very quickly. | ① | ② | ③ | ④ | ⑤ |
| 1. I found the BloG3 app very cumbersome to use. | ① | ② | ③ | ④ | ⑤ |
| 1. I felt very confident using the BloG3 app. | ① | ② | ③ | ④ | ⑤ |
| 1. I had to learn a lot of things before I could get started with the BloG3 app. | ① | ② | ③ | ④ | ⑤ |

**BLOCK F**

**Do you see any particular advantages compared to current patient care when using the BloG3 app?**

**Do you see any specific challenges when using the BloG3 app?**

**Do you have any comments or suggestions for improvement regarding the BloG3 app?**

**Thank you for your time!**
