# Supplementary material for: Patient Perceptions of Blockchain-Based Health Information Exchange: User-Centered Design Study
Source: J Med Internet Res. 2026 Mar 11;28:e78849. doi: 10.2196/78849 (PMC13000691; doi:10.2196/78849)
Supplement: Multimedia Appendix 5 [file jmir-v28-e78849-s005.docx]

**Supplementary Table.** Phase 3 Questions and Patient responses.

| **Variables** | **Participants (n=32)** |
| --- | --- |
|  |  |
| **Task 1: Connection to Institution [1. Using the BloG³ app improves my performance when completing such a task. ]** |  |
| Strongly agree | 11 (34.38%) |
| Agree | 8 (25.00%) |
| Somewhat agree | 2 (6.25%) |
| Neutral | 9 (28.13%) |
| Somewhat disagree | 1 (3.13%) |
| Disagree | 1 (3.13%) |
| Strongly Disagree | 0 (0.00%) |
| **Task 1: Connection to Institution [2. Using the BloG³ app improves my productivity when completing such a task. ]** |  |
| Strongly agree | 11 (34.38%) |
| Agree | 8 (25.00%) |
| Somewhat agree | 2 (6.25%) |
| Neutral | 9 (28.13%) |
| Somewhat disagree | 1 (3.13%) |
| Disagree | 1 (3.13%) |
| Strongly Disagree | 0 (0.00%) |
| **Task 1: Connection to Institution [3. Using the BloG³ app improves my effectiveness when completing such a task. ]** |  |
| Strongly agree | 13 (40.63%) |
| Agree | 5 (15.63%) |
| Somewhat agree | 2 (6.25%) |
| Neutral | 9 (28.13%) |
| Somewhat disagree | 1 (3.13%) |
| Disagree | 1 (3.13%) |
| Strongly Disagree | 1 (3.13%) |
| **Task 1: Connection to Institution [4. I find the BloG³ app useful for completing such a task. ]** |  |
| Strongly agree | 15 (46.88%) |
| Agree | 3 (9.38%) |
| Somewhat agree | 5 (15.63%) |
| Neutral | 6 (18.75%) |
| Somewhat disagree | 1 (3.13%) |
| Disagree | 2 (6.25%) |
| Strongly Disagree | 0 (0.00%) |

| **Task 1: Connection to Institution [5. Usage of the BloG³ app is clear and understandable when completing such a task. ]** |  |
| --- | --- |
| Strongly agree | 12 (37.50%) |
| Agree | 4 (12.50%) |
| Somewhat agree | 4 (12.50%) |
| Neutral | 7 (21.88%) |
| Somewhat disagree | 3 (9.38%) |
| Disagree | 2 (6.25%) |
| Strongly Disagree | 0 (0.00%) |
| **Task 1: Connection to Institution [6. Usage of the BloG³ app doesn’t require much cognitive effort when completing such a task. ]** |  |
| Strongly agree | 12 (37.50%) |
| Agree | 7 (21.88%) |
| Somewhat agree | 5 (15.63%) |
| Neutral | 6 (18.75%) |
| Somewhat disagree | 0 (0.00%) |
| Disagree | 2 (6.25%) |
| Strongly Disagree | 0 (0.00%) |
| **Task 1: Connection to Institution [7. I find the BloG³ App easy to use when completing such a task. ]** |  |
| Strongly agree | 13 (40.63%) |
| Agree | 6 (18.75%) |
| Somewhat agree | 4 (12.50%) |
| Neutral | 6 (18.75%) |
| Somewhat disagree | 2 (6.25%) |
| Disagree | 2 (6.25%) |
| Strongly Disagree | 0 (0.00%) |
| **Task 1: Connection to Institution [8. When completing such a task, I find it easy to get the BloG³ App to do what I want. ]** |  |
| Strongly agree | 11 (34.38%) |
| Agree | 5 (15.63%) |
| Somewhat agree | 7 (21.88%) |
| Neutral | 7 (21.88%) |
| Somewhat disagree | 0 (0.00%) |
| Disagree | 2 (6.25%) |
| Strongly Disagree | 0 (0.00%) |
| **Task 1: Connection to Institution [9. Assuming I had access to the BloG³ App, I would intend to use it. ]** |  |
| Strongly agree | 13 (40.63%) |
| Agree | 7 (21.88%) |
| Somewhat agree | 3 (9.38%) |
| Neutral | 7 (21.88%) |
| Somewhat disagree | 0 (0.00%) |
| Disagree | 1 (3.13%) |
| Strongly Disagree | 1 (3.13%) |
| **Task 1: Connection to Institution [10. Assuming I had access to the BloG³ App, I predict that I would use it. ]** |  |
| Strongly agree | 12 (37.50%) |
| Agree | 9 (28.13%) |
| Somewhat agree | 2 (6.25%) |
| Neutral | 7 (21.88%) |
| Somewhat disagree | 0 (0.00%) |
| Disagree | 2 (6.25%) |
| Strongly Disagree | 0 (0.00%) |
| **Task 2: Sharing Documents [1. Using the BloG³ app improves my performance when completing such a task.]** |  |
| Strongly agree | 12 (37.50%) |
| Agree | 11 (34.38%) |
| Somewhat agree | 3 (9.38%) |
| Neutral | 5 (15.63%) |
| Somewhat disagree | 0 (0.00%) |
| Disagree | 1 (3.13%) |
| Strongly Disagree | 0 (0.00%) |
| **Task 2: Sharing Documents [2. Using the BloG³ app improves my productivity when completing such a task. ]** |  |
| Strongly agree | 13 (40.63%) |
| Agree | 10 (31.25%) |
| Somewhat agree | 0 (0.00%) |
| Neutral | 6 (18.75%) |
| Somewhat disagree | 0 (0.00%) |
| Disagree | 3 (9.38%) |
| Strongly Disagree | 0 (0.00%) |
| **Task 2: Sharing Documents [3. Using the BloG³ app improves my effectiveness when completing such a task.]** |  |
| Strongly agree | 13 (40.63%) |
| Agree | 10 (31.25%) |
| Somewhat agree | 3 (9.38%) |
| Neutral | 5 (15.63%) |
| Somewhat disagree | 0 (0.00%) |
| Disagree | 1 (3.13%) |
| Strongly Disagree | 0 (0.00%) |
| **Task 2: Sharing Documents [4. I find the BloG³ app useful for completing such a task.]** |  |
| Strongly agree | 14 (43.75%) |
| Agree | 10 (31.25%) |
| Somewhat agree | 3 (9.38%) |
| Neutral | 4 (12.50%) |
| Somewhat disagree | 0 (0.00%) |
| Disagree | 1 (3.13%) |
| Strongly Disagree | 0 (0.00%) |
| **Task 2: Sharing Documents [5.** **Usage of the BloG³ app is clear and understandable when completing such a task. ]** |  |
| Strongly agree | 13 (40.63%) |
| Agree | 10 (31.25%) |
| Somewhat agree | 2 (6.25%) |
| Neutral | 2 (6.25%) |
| Somewhat disagree | 2 (6.25%) |
| Disagree | 3 (9.38%) |
| Strongly Disagree | 0 (0.00%) |
| **Task 2: Sharing Documents [6.** **Usage of the BloG³ app doesn’t require much cognitive effort when completing such a task. ]** |  |
| Strongly agree | 12 (37.50%) |
| Agree | 11 (34.38%) |
| Somewhat agree | 3 (9.38%) |
| Neutral | 3 (9.38%) |
| Somewhat disagree | 0 (0.00%) |
| Disagree | 3 (9.38%) |
| Strongly Disagree | 0 (0.00%) |
| **Task 2: Sharing Documents [7.** **I find the BloG³ App easy to use when completing such a task. ]** |  |
| Strongly agree | 12 (37.50%) |
| Agree | 10 (31.25%) |
| Somewhat agree | 5 (15.63%) |
| Neutral | 3 (9.38%) |
| Somewhat disagree | 0 (0.00%) |
| Disagree | 2 (6.25%) |
| Strongly Disagree | 0 (0.00%) |
| **Task 2: Sharing Documents [8. When completing such a task, I find it easy to get the BloG³ App to do what I want. ]** |  |
| Strongly agree | 10 (31.25%) |
| Agree | 11 (34.38%) |
| Somewhat agree | 4 (12.50%) |
| Neutral | 4 (12.50%) |
| Somewhat disagree | 1 (3.13%) |
| Disagree | 2 (6.25%) |
| Strongly Disagree | 0 (0.00%) |
| **Task 2: Sharing Documents [9. Assuming I had access to the BloG³ App, I would intend to use it. ]** |  |
| Strongly agree | 13 (40.63%) |
| Agree | 10 (31.25%) |
| Somewhat agree | 1 (3.13%) |
| Neutral | 6 (18.75%) |
| Somewhat disagree | 0 (0.00%) |
| Disagree | 1 (3.13%) |
| Strongly Disagree | 1 (3.13%) |
| **Task 2: Sharing Documents [10. Assuming I had access to the BloG³ App, I predict that I would use it. ]** |  |
| Strongly agree | 12 (37.50%) |
| Agree | 11 (34.38%) |
| Somewhat agree | 1 (3.13%) |
| Neutral | 5 (15.63%) |
| Somewhat disagree | 0 (0.00%) |
| Disagree | 2 (6.25%) |
| Strongly Disagree | 1 (3.13%) |
| **Task 3: Diary Entries [1. Using the BloG³ app improves my performance when completing such a task. ]** |  |
| Strongly agree | 11 (34.38%) |
| Agree | 10 (31.25%) |
| Somewhat agree | 4 (12.50%) |
| Neutral | 6 (18.75%) |
| Somewhat disagree | 0 (0.00%) |
| Disagree | 0 (0.00%) |
| Strongly Disagree | 1 (3.13%) |
| **Task 3: Diary Entries [2. Using the BloG³ app improves my productivity when completing such a task. ]** |  |
| Strongly agree | 12 (37.50%) |
| Agree | 8 (25.00%) |
| Somewhat agree | 4 (12.50%) |
| Neutral | 7 (21.88%) |
| Somewhat disagree | 0 (0.00%) |
| Disagree | 1 (3.13%) |
| Strongly Disagree | 0 (0.00%) |
| **Task 3: Diary Entries [3. Using the BloG³ app improves my effectiveness when completing such a task. ]** |  |
| Strongly agree | 12 (37.50%) |
| Agree | 8 (25.00%) |
| Somewhat agree | 4 (12.50%) |
| Neutral | 7 (21.88%) |
| Somewhat disagree | 0 (0.00%) |
| Disagree | 1 (3.13%) |
| Strongly Disagree | 0 (0.00%) |
| **Task 3: Diary Entries [4. I find the BloG³ app useful for completing such a task. ]** |  |
| Strongly agree | 12 (37.50%) |
| Agree | 10 (31.25%) |
| Somewhat agree | 5 (15.63%) |
| Neutral | 4 (0.00%) |
| Somewhat disagree | 0 (0.00%) |
| Disagree | 1 (3.13%) |
| Strongly Disagree | 0 (0.00%) |
| **Task 3: Diary Entries [5. Usage of the BloG³ app is clear and understandable when completing such a task. ]** |  |
| Strongly agree | 11 (34.38%) |
| Agree | 12 (37.50%) |
| Somewhat agree | 1 (3.13%) |
| Neutral | 4 (12.50%) |
| Somewhat disagree | 2 (6.25%) |
| Disagree | 2 (6.25%) |
| Strongly Disagree | 0 (0.00%) |
| **Task 3: Diary Entries [6.** **Usage of the BloG³ app doesn’t require much cognitive effort when completing such a task. ]** |  |
| Strongly agree | 11 (34.38%) |
| Agree | 10 (31.25%) |
| Somewhat agree | 2 (6.25%) |
| Neutral | 6 (18.75%) |
| Somewhat disagree | 0 (0.00%) |
| Disagree | 3 (9.38%) |
| Strongly Disagree | 0 (0.00%) |
| **Task 3: Diary Entries [7. I find the BloG³ App easy to use when completing such a task. ]** |  |
| Strongly agree | 11 (34.38%) |
| Agree | 10 (31.25%) |
| Somewhat agree | 5 (15.63%) |
| Neutral | 5 (15.63%) |
| Somewhat disagree | 0 (0.00%) |
| Disagree | 1 (3.13%) |
| Strongly Disagree | 0 (0.00%) |
| **Task 3: Diary Entries [8. When completing such a task, I find it easy to get the BloG³ App to do what I want. ]** |  |
| Strongly agree | 10 (31.25%) |
| Agree | 12 (37.50%) |
| Somewhat agree | 5 (15.63%) |
| Neutral | 4 (12.50%) |
| Somewhat disagree | 0 (0.00%) |
| Disagree | 1 (3.13%) |
| Strongly Disagree | 0 (0.00%) |
| **Task 3: Diary Entries [9. Assuming I had access to the BloG³ App, I would intend to use it. ]** |  |
| Strongly agree | 13 (40.63%) |
| Agree | 7 (21.88%) |
| Somewhat agree | 3 (9.38%) |
| Neutral | 5 (15.63%) |
| Somewhat disagree | 1 (3.13%) |
| Disagree | 2 (6.25%) |
| Strongly Disagree | 1 (3.13%) |
| **Task 3: Diary Entries [10. Assuming I had access to the BloG³ App, I predict that I would use it. ]** |  |
| Strongly agree | 11 (34.38%) |
| Agree | 10 (31.25%) |
| Somewhat agree | 3 (9.38%) |
| Neutral | 3 (9.38%) |
| Somewhat disagree | 0 (0.00%) |
| Disagree | 5 (15.63%) |
| Strongly Disagree | 0 (0.00%) |
| **Task 4: Nursing Service Discovery [1. Using the BloG³ app improves my performance when completing such a task. ]** |  |
| Strongly agree | 8 (25.00%) |
| Agree | 12 (37.50%) |
| Somewhat agree | 3 (9.38%) |
| Neutral | 8 (25.00%) |
| Somewhat disagree | 0 (0.00%) |
| Disagree | 1 (3.13%) |
| Strongly Disagree | 0 (0.00%) |
| **Task 4: Nursing Service Discovery [2. Using the BloG³ app improves my productivity when completing such a task.. ]** |  |
| Strongly agree | 8 (25.00%) |
| Agree | 11 (34.38%) |
| Somewhat agree | 4 (12.50%) |
| Neutral | 8 (25.00%) |
| Somewhat disagree | 1 (3.13%) |
| Disagree | 0 (0.00%) |
| Strongly Disagree | 0 (0.00%) |
| **Task 4: Nursing Service Discovery [3. Using the BloG³ app improves my effectiveness when completing such a task. ]** |  |
| Strongly agree | 8 (25.00%) |
| Agree | 12 (37.50%) |
| Somewhat agree | 3 (9.38%) |
| Neutral | 8 (25.00%) |
| Somewhat disagree | 1 (3.13%) |
| Disagree | 0 (0.00%) |
| Strongly Disagree | 0 (0.00%) |
| **Task 4: Nursing Service Discovery [4. I find the BloG³ app useful for completing such a task. ]** |  |
| Strongly agree | 8 (25.00%) |
| Agree | 13 (40.63%) |
| Somewhat agree | 5 (15.63%) |
| Neutral | 6 (18.75%) |
| Somewhat disagree | 0 (0.00%) |
| Disagree | 0 (0.00%) |
| Strongly Disagree | 0 (0.00%) |
| **Task 4: Nursing Service Discovery [5. Usage of the BloG³ app is clear and understandable when completing such a task. ]** |  |
| Strongly agree | 7 (21.88%) |
| Agree | 15 (46.88%) |
| Somewhat agree | 5 (15.63%) |
| Neutral | 4 (12.50%) |
| Somewhat disagree | 0 (0.00%) |
| Disagree | 0 (0.00%) |
| Strongly Disagree | 1 (3.13%) |
| **Task 4: Nursing Service Discovery [6.** **Usage of the BloG³ app doesn’t require much cognitive effort when completing such a task. ]** |  |
| Strongly agree | 8 (25.00%) |
| Agree | 13 (40.63%) |
| Somewhat agree | 4 (12.50%) |
| Neutral | 7 (21.88%) |
| Somewhat disagree | 0 (0.00%) |
| Disagree | 0 (0.00%) |
| Strongly Disagree | 0 (0.00%) |
| **Task 4: Nursing Service Discovery [7. I find the BloG³ App easy to use when completing such a task. ]** |  |
| Strongly agree | 8 (25.00%) |
| Agree | 12 (37.50%) |
| Somewhat agree | 5 (15.63%) |
| Neutral | 6 (18.75%) |
| Somewhat disagree | 0 (0.00%) |
| Disagree | 0 (0.00%) |
| Strongly Disagree | 1 (3.13%) |
| **Task 4: Nursing Service Discovery [8. When completing such a task, I find it easy to get the BloG³ App to do what I want. ]** |  |
| Strongly agree | 7 (21.88%) |
| Agree | 13 (40.63%) |
| Somewhat agree | 4 (12.50%) |
| Neutral | 6 (18.75%) |
| Somewhat disagree | 1 (3.13%) |
| Disagree | 1 (3.13%) |
| Strongly Disagree | 0 (0.00%) |
| **Task 4: Nursing Service Discovery [9. Assuming I had access to the BloG³ App, I would intend to use it. ]** |  |
| Strongly agree | 8 (25.00%) |
| Agree | 12 (37.50%) |
| Somewhat agree | 4 (12.50%) |
| Neutral | 7 (21.88%) |
| Somewhat disagree | 0 (0.00%) |
| Disagree | 0 (0.00%) |
| Strongly Disagree | 1 (3.13%) |
| **Task 4: Nursing Service Discovery [10. Assuming I had access to the BloG³ App, I predict that I would use it. ]** |  |
| Strongly agree | 6 (18.75%) |
| Agree | 14 (43.75%) |
| Somewhat agree | 3 (9.38%) |
| Neutral | 7 (21.88%) |
| Somewhat disagree | 0 (0.00%) |
| Disagree | 2 (6.25%) |
| Strongly Disagree | 0 (0.00%) |
| **SUS [1. I think I would like using the BlOG^3^ App often. ]** |  |
| Strongly agree | 12 (37.50%) |
| Agree | 11 (34.38%) |
| Somewhat agree | 0 (0.00%) |
| Neutral | 6 (18.75%) |
| Somewhat disagree | 0 (0.00%) |
| Disagree | 1 (3.13%) |
| Strongly Disagree | 2 (6.25%) |
| **SUS [2. I found the BlOG^3^ App to be unnecessarily complex. ]** |  |
| Strongly agree | 0 (0.00%) |
| Agree | 2 (6.25%) |
| Somewhat agree | 0 (0.00%) |
| Neutral | 4 (12.50%) |
| Somewhat disagree | 0 (0.00%) |
| Disagree | 11 (34.38%) |
| Strongly Disagree | 15 (46.88%) |
| **SUS [3. I thought the BlOG^3^ App was easy to operate. ]** |  |
| Strongly agree | 10 (31.25%) |
| Agree | 10 (31.25%) |
| Somewhat agree | 0 (0.00%) |
| Neutral | 5 (15.63%) |
| Somewhat disagree | 0 (0.00%) |
| Disagree | 5 (15.63%) |
| Strongly Disagree | 2 (6.25%) |
| **SUS [4. I think that I need the help of a technically versed person to use the BlOG^3^ App. ]** |  |
| Strongly agree | 2 (6.25%) |
| Agree | 2 (6.25%) |
| Somewhat agree | 0 (0.00%) |
| Neutral | 4 (12.5%) |
| Somewhat disagree | 0 (0.00%) |
| Disagree | 7 (21.88%) |
| Strongly Disagree | 17 (53.13%) |
| **SUS [5. I found that the different functions were well integrated into the BlOG^3^ App. ]** |  |
| Strongly agree | 11 (34.38%) |
| Agree | 13 (40.63%) |
| Somewhat agree | 0 (0.00%) |
| Neutral | 7 (21.88%) |
| Somewhat disagree | 0 (0.00%) |
| Disagree | 0 (0.00%) |
| Strongly Disagree | 1 (3.13%) |
| **SUS [6. I found the BlOG^3^ App to not be sufficiently consistent. ]** |  |
| Strongly agree | 0 (0.00%) |
| Agree | 1 (3.13%) |
| Somewhat agree | 0 (0.00%) |
| Neutral | 14 (43.75%) |
| Somewhat disagree | 0 (0.00%) |
| Disagree | 6 (18.75%) |
| Strongly Disagree | 11 (34.38%) |
| **SUS [7. I would imagine that most people would be able to learn to use the BlOG^3^ App very quickly. ]** |  |
| Strongly agree | 15 (46.88%) |
| Agree | 10 (31.25%) |
| Somewhat agree | 0 (0.00%) |
| Neutral | 5 (15.63%) |
| Somewhat disagree | 0 (0.00%) |
| Disagree | 0 (0.00%) |
| Strongly Disagree | 2 (6.25%) |
| **SUS [8. I found using the BlOG^3^ App to be very cumbersome. ]** |  |
| Strongly agree | 0 (0.00%) |
| Agree | 3 (9.38%) |
| Somewhat agree | 0 (0.00%) |
| Neutral | 5 (15.63%) |
| Somewhat disagree | 0 (0.00%) |
| Disagree | 11 (34.38%) |
| Strongly Disagree | 13 (40.63%) |
| **SUS [9. I felt very confident using the BlOG^3^ App. ]** |  |
| Strongly agree | 12 (37.50%) |
| Agree | 11 (34.38%) |
| Somewhat agree | 0 (0.00%) |
| Neutral | 7 (21.88%) |
| Somewhat disagree | 0 (0.00%) |
| Disagree | 1 (3.13%) |
| Strongly Disagree | 1 (3.13%) |

| **SUS [10. I had to learn many things before being able to begin using the BlOG^3^ App. ]** |  |
| --- | --- |
| Strongly agree | 0 (0.00%) |
| Agree | 2 (6.25%) |
| Somewhat agree | 0 (0.00%) |
| Neutral | 7 (21.88%) |
| Somewhat disagree | 0 (0.00%) |
| Disagree | 8 (25.00%) |
| Strongly Disagree | 15 (46.88%) |
