# Supplementary material for: Patient Perceptions of Blockchain-Based Health Information Exchange: User-Centered Design Study
Source: J Med Internet Res. 2026 Mar 11;28:e78849. doi: 10.2196/78849 (PMC13000691; doi:10.2196/78849)
Supplement: Multimedia Appendix 6 [file jmir-v28-e78849-s006.docx]

To examine whether participants’ gender and age influenced their ratings for PEOU, PU, and BI for all four tasks and their SUS rating in phase 3, we performed a subgroup analysis using Aligned Rank Transform (ART) ANOVAs. Each model included participants’ gender (male, female), age group (older and younger than 59), and their interaction (gender × age) as fixed factors. Moreover, we extended our analysis by using two ART one-way ANOVAs for SUS with the number and frequency IT systems used for document management as separated fixed factors. To examine the differences in SUS we divided participants into two groups (0 = low use, 1 = high use) for both fixed factors. Note, given the limited sample size, the results of all ART ANOVAs should be interpreted with care. We recommend future research to replicate our study with a larger sample.

The ART ANOVA indicated that gender had a significant effect on the participant ratings of PEOU for tasks 1 to 3 and on the ratings of BI for tasks 1 and 2 (see Table 1). Due to the slight imbalance between female (n=19) and male (n=13) participants, the results should be interpreted with caution. Neither the effects of age groups (older and younger than 59), nor the interaction effect of age and gender was significant for any task.

**Table 1.** ANOVA results for TAM for all four tasks of phase 3 by gender and age.

| Task | Construct | Factor | *F* value | df | *P* value | Statistical Significance |
| --- | --- | --- | --- | --- | --- | --- |
| 1 | BI^a^ | Gender | 4.585 | 1.28 | 0.041 | **Yes** |
|  |  | Age Group | 0.605 | 1.28 | 0.443 | No |
|  |  | Gender × Age Group | 0.409 | 1.28 | 0.528 | No |
|  | PU^b^ | Gender | 2.875 | 1.28 | 0.101 | No |
|  |  | Age Group | 0.034 | 1.28 | 0.855 | No |
|  |  | Gender × Age Group | 0.014 | 1.28 | 0.908 | No |
|  | PEOU^c^ | Gender | 7.735 | 1.28 | 0.01 | **Yes** |
|  |  | Age Group | 0.644 | 1.28 | 0.429 | No |
|  |  | Gender × Age Group | 0.425 | 1.28 | 0.52 | No |
| 2 | BI | Gender | 12.084 | 1.28 | 0.002 | **Yes** |
|  |  | Age Group | 0.266 | 1.28 | 0.61 | No |
|  |  | Gender × Age Group | 0,001 | 1.28 | 0.98 | No |
|  | PU | Gender | 3.345 | 1.28 | 0.078 | No |
|  |  | Age Group | 0.01 | 1.28 | 0.92 | No |
|  |  | Gender × Age Group | 0.585 | 1.28 | 0.451 | No |
|  | PEOU | Gender | 8.191 | 1.28 | 0.008 | **Yes** |
|  |  | Age Group | 0.463 | 1.28 | 0.502 | No |
|  |  | Gender × Age Group | 0.494 | 1.28 | 0.488 | No |
| 3 | BI | Gender | 2.845 | 1.28 | 0.103 | No |
|  |  | Age Group | 0.075 | 1.28 | 0,787 | No |
|  |  | Gender × Age Group | 0.651 | 1.28 | 0.427 | No |
|  | PU | Gender | 1.71 | 1.28 | 0.202 | No |
|  |  | Age Group | 0.656 | 1.28 | 0.425 | No |
|  |  | Gender × Age Group | 0.316 | 1.28 | 0.578 | No |
|  | PEOU | Gender | 4.334 | 1.28 | 0.047 | **Yes** |
|  |  | Age Group | 0.468 | 1.28 | 0.499 | No |
|  |  | Gender × Age Group | 0.31 | 1.28 | 0.582 | No |
| 4 | BI | Gender | 0.514 | 1.28 | 0.479 | No |
|  |  | Age Group | 0.301 | 1.28 | 0.588 | No |
|  |  | Gender × Age Group | 0.455 | 1.28 | 0.506 | No |
|  | PU | Gender | 3.235 | 1.28 | 0.083 | No |
|  |  | Age Group | 0.881 | 1.28 | 0.356 | No |
|  |  | Gender × Age Group | 0.173 | 1.28 | 0.681 | No |
|  | PEOU | Gender | 1.671 | 1.28 | 0.207 | No |
|  |  | Age Group | 0.219 | 1.28 | 0.643 | No |
|  |  | Gender × Age Group | 0.061 | 1.28 | 0.806 | No |

^a^BI: Behavioral Intention to use.

^b^PU: Perceived Usefulness.

^c^PEOU: Perceived Ease of Use.

The ART ANOVA for SUS did only show a slight effect (*F*(1,28) = 2.54, *p* = .122) of age on SUS (see Table 2). The BC-based HIE mobile application received a higher SUS score of 84.12 among female participants. In contrast, male participant provided a rating of 67.43 (graded as “C”). The ART ANOVA confirmed that female participants gave higher SUS ratings to the BC-based HIE mobile application than male participants (*F*(1,28) = 6.00, *p* = .021). Given the slight imbalance in gender distribution (female: n=19, male: n=13), the effects of age and gender should be interpreted with caution. The additional one-way ART ANOVA performed did not show a significant effect of the number (F(1, 30) = 0.668, p = 0.420) or the frequency (F(1, 30) = 0.338, p = 0.566) of document management systems used on the SUS.

**Table 2.** ANOVA results for SUS scores in phase 3 by gender and age.

| Task | Construct | Factor | *F* value | df | *P* value | Statistical Significance |
| --- | --- | --- | --- | --- | --- | --- |
| Overall App | SUS^a^ | Gender | 6.004 | 1.28 | 0.021 | Yes |
|  | SUS | Age Group | 2.539 | 1.28 | 0.122 | No |
|  | SUS | Gender × Age Group | 1.502 | 1.28 | 0.231 | No |

^a^System Usability Scale.
